# Subnanometer Tracking of the Oxidation State on Co_3_O_4_ Nanoparticles by Identical Location Imaging and Spectroscopy

**DOI:** 10.1021/acsami.4c20690

**Published:** 2025-01-31

**Authors:** Franz-Philipp Schmidt, Thomas Götsch, Sharif Najafishirtari, Malte Behrens, Christoph Pratsch, Stephane Kenmoe, Dick Hartmann Douma, Frank Girgsdies, Jasmin Allan, Axel Knop-Gericke, Thomas Lunkenbein

**Affiliations:** † Department of Inorganic Chemistry, Fritz-Haber-Institut der Max-Planck-Gesellschaft, Berlin 14195, Germany; ‡ Institute of Inorganic Chemistry, Christian-Albrechts-Universität zu Kiel, Kiel 24118, Germany; § Kiel Nano, Surface and Interface Science KiNSIS, Christian-Albrechts-Universität zu Kiel, Kiel 24118, Germany; ∥ Department X-Ray Microscopy, Helmholtz-Zentrum Berlin für Materialien und Energie GmbH, Berlin 12489, Germany; ⊥ Department of Theoretical Chemistry, University of Duisburg-Essen, Essen 45141, Germany; # Faculté des Sciences et Techniques, Groupe de Simulations Numériques en Magnétisme et Catalyse, Université Marien Ngouabi, Brazzaville B.P. 69, Congo; ¶ Department of Heterogeneous Reactions, Max Planck Institute for Chemical Energy Conversion, Mülheim 45470, Germany

**Keywords:** ILIAS, quasi in situ electron microscopy, TEM, EELS, catalysis, Co_3_O_4_, oxidation state

## Abstract

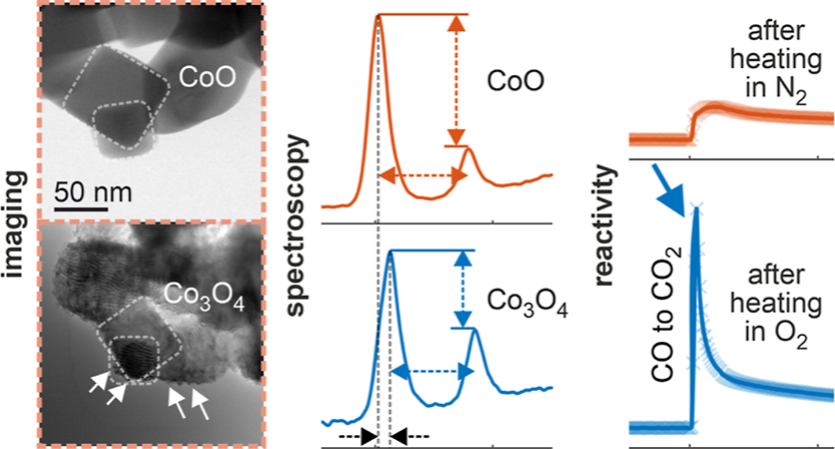

Understanding a catalytic reaction requires tools that elucidate the structure of the catalyst surface and subsurface, ideally at atomic resolution and under reaction conditions. Operando electron microscopy meets this requirement in some cases, but fails in others where the required reaction conditions cannot be reached or lead to an unwanted influence of the electron beam on the reactant and catalyst. We introduce ILIAS (identical location imaging and spectroscopy) in combination with a quasi in situ approach to disentangle the effect of heat and gas on the surface of nanoparticles from the effect of the electron beam. With this approach we allow high temperatures and pressures in any gaseous environment on the one hand, and atomic resolution imaging and spectroscopy on the other. As a proof of concept, we resolve the structural evolution of a Co_3_O_4_ spinel catalyst using ILIAS and track the oxidation state across the surface before and after heating in a reductive or oxidative environment. We then titrate the surface of the catalyst using CO as a probe molecule to remove highly active oxygen species formed during the thermal treatment, providing unprecedented insight into the interplay between pretreatment and surface reactivity of Co_3_O_4_ nanoparticles.

## 1 Introduction

A major goal in catalysis research is to establish a correlation between the structure of a catalyst and its catalytic properties in order to understand how this catalyst works.^1^ This is a challenging task, mainly because the definition of the problem itself is very vague for two reasons: What is meant by structure? And what is meant by the catalytic properties? In heterogeneous catalysis, reference is often made to the geometric structure of a crystalline particle, in particular to the uppermost atomic layers and their specific surface termination, which influence the catalytic reaction.^2−4^ However, this is an oversimplification, since, in addition to other factors, not only the geometric structure but also the electronic structure play a role.^5^ The most relevant catalytic property, in turn, is understood as the ability of a catalyst to efficiently convert a reactant into the desired product.^6^ The conversion can be a highly complex multistep process, often accompanied by undesirable side reactions. Therefore, it is difficult to relate a specific structural feature of the catalyst to the product formation in a catalytic reaction.

Decoupling of the involved steps can facilitate the identification of structural features of a catalyst that are potentially relevant for product formation. On the one hand, high spatial resolution techniques such as (scanning) transmission electron microscopy ((S)TEM) can image the catalyst surface with atomic resolution.^7−10^ On the other hand, electron energy loss spectrometry (EELS) can map the electronic structure of the catalyst surface.^11,12^ Combining both techniques in the same experiment (STEM-EELS) provides simultaneous access to the geometric structure—which includes the crystal structure, defects, surface termination and composition^13^—as well as the electronic structure of the catalytic particle and its surfaces. This combined technique already helps to partially disentangle the problem, as both geometric and electronic structural changes can be tracked separately but within the same experiment at the same location of the catalyst. However, for STEM-EELS applied in operando setups,^14^ further issues come into play, that often prevent conclusive results. First, this is because the required operando conditions can limit the generation of a significant signal-to-noise ratio. Increased scattering because of high pressures and temperatures hinder a clear interpretation, in particular for EELS. Second, the unwanted influence of the electron beam on the reactant and catalyst can falsify the result in terms of increased beam damage compared to conventional electron microscopy. Instead of using operando electron microscopy, a quasi in situ approach, which allows the targeted use of various gases, pressures, flows and temperatures in a dedicated microreactor, helps to combine high pressures and high temperatures and to reduce beam damage, although the price to be paid is the insensitivity of this method to detect metastable states.

In this work, we present ILIAS—identical location imaging and spectroscopy—an approach to track the evolution of the geometric and electronic structure of oxide nanoparticles exposed to different reaction parameters. As a proof of concept we study a Co_3_O_4_ spinel, which is often applied as a model system in thermal and electrocatalytic reactions.^15−17^ We combine ILIAS with quasi in situ electron microscopy^18^ and compare structural changes as a function of different pretreatment with the ability to oxidize CO as a probe reaction. Pretreatment means heating in different gases, namely in oxidative (O_2_) or reductive/inert (N_2_ or He) gases at mild temperatures ranging from room temperature (RT) to 250 °C. In this way, the structural changes and the evolution across the surface (surface/subsurface/bulk) resulting only from the pretreatment are followed before and after heating and are compared to the output of the subsequent surface titration of the nanoparticles using CO as a probe molecule to form CO_2_. The observed differences in CO_2_ formation are dependent on structural (geometric and electronic) features created during the pretreatment. Importantly, these features are resolved with subnanometer precision, i.e. at the scale at which the surface reactions occur.

In addition to high resolution imaging, we are placing emphasis on the spectral evolution (change in oxidation state) across the surface with subnanometer resolution using EELS.^19^ This also requires the development of postprocessing tools to extract relevant signals from EEL spectra to derive spatially resolved values for the oxidation state. These results will be compared with those obtained using X-ray techniques, such as near ambient pressure X-ray photoelectron spectroscopy (NAP-XPS) and transmission X-ray microscopy (TXM), and are complemented by density functional theory (DFT) simulations.

## 2 Results and Discussion

### 2.1 ILIAS and Quasi In Situ Setup

**Figure 1:**
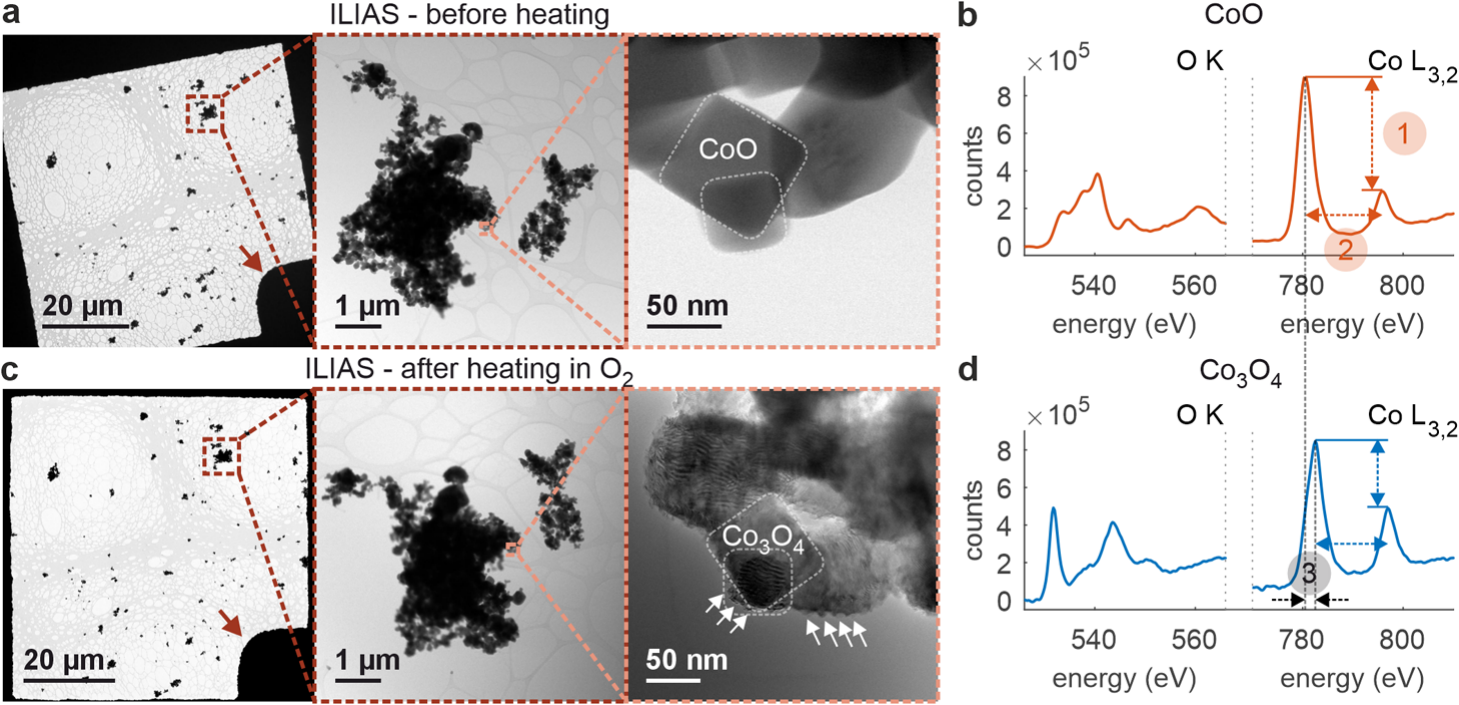
Identical location imaging and spectroscopy. (a) ILIAS before heating in O_2_. TEM micrographs at different magnifications, from overview (left) to medium (center) to high (right). The small dashed rectangles in the left and middle images indicate the enlarged regions shown in the middle and right panels, respectively. (b) EEL spectra of the particle labeled “CoO” in the right image of (a). (c) ILIAS after heating in O_2_ of the same regions as in (a). The white arrows in the right image highlight the strong restructuring at the surface of the particles. (d) The corresponding EEL spectra of a Co_3_O_4_ particle after heating in O_2_. The differences in peak ratio, energy difference and peak position are labeled 1, 2 and 3.

ILIAS in combination with quasi in situ electron microscopy (Figure 1) allows the study of the same nanoparticle before and after reaction (i.e., heating in a defined gas mixture, pressure and flow). The reaction is performed outside the microscope in a self-made TEM grid microreactor, which is connected to the quasi in situ setup shown in the Supporting Information in Figures S1 and S2. The TEM grid with the sample is transferred between the microreactor and the microscope under inert conditions. Thus, the sample can be analyzed without contact to ambient air. This approach has the advantage over spatial averaging techniques, because structural changes at the atomic scale of the same particle or the same surface can be correlated with different heating and gas treatments. In contrast to operando techniques, the heat effect and gas reaction on the surface are decoupled from a potential influence by the electron beam. A disadvantage compared to operando techniques is that metastable states, which only exist during heating and gas treatment, cannot be detected. It is important to note that the more realistic reaction parameters in this quasi in situ setup can be achieved (e.g., higher pressure) compared to existing closed and open cell operando TEM setups.^20−24^ This allows reaction-relevant conditions to bridge the pressure gap,^25^ which is difficult for many operando techniques.

Figure 1 illustrates the result of ILIAS for a powder sample investigated in this work. It consists of Co_3_O_4_ spinel nanoparticles (see Rietveld refined X-ray diffractograms in Figure S3 in the Supporting Information), including a minor fraction of CoO particles. Figure 1a shows the sample before heating in O_2_. In the right panel of Figure 1a, two particles are surrounded by white dashed lines, which we identify as CoO particles with the help of EELS (Figure 1b). The energy loss near edge structure (ELNES) of the O K- and Co L_3,2_-edge has a specific shape which is typical for CoO^26^ and clearly differs from cobalt oxides of other oxidation states.^27^ The sample was then heated in the quasi in situ setup in O_2_ at 250 °C (see Methods) and transferred back to the TEM using an inert sample transfer holder. Figure 1c presents TEM images of the same position and the same particles after heating in O_2_. The red arrow in the left image points to the center of the TEM grid [compare with the left image in (a)], while the white arrows in the right image highlight the strong restructuring at the surface of the particles [compare with the right image in (a)]. This restructuring indicates modification of the cobalt oxide due to heating in O_2_, but does not allow to conclude on the final oxidation state of the cobalt oxide particle. Conventional identical location imaging (ILI)^28^ would therefore be insufficient, but by combining imaging with spectroscopy (ILIAS) we could verify the phase transition from CoO to Co_3_O_4_ (Figure 1d). While the ELNES of the O K-edge also has a distinct shape (e.g., the appearance of the prepeak at 532 eV, followed by a different peak composition),^29,30^ we concentrate in the following on the ELNES of the Co L_3,2_-edge. We focus on three features: The L_3_/L_2_ peak ratio (1), the L_3_-L_2_ peak energy difference (2) and the L_3_ peak maximum position (3). Comparing these three features before and after heating in O_2_, we observe a decrease in the peak ratio (1), a decrease in the peak energy difference (2) and an increase in the L_3_ peak energy (3). These results are in good agreement with the reference spectra for CoO and Co_3_O_4_. The combination of imaging and spectroscopy allows the unambiguous assignment of the oxidation state at the very local scale of the same particle before and after heating.

### 2.2 Surface Reactivity

Co_3_O_4_ is composed of divalent (Co^2+^) and trivalent (Co^3+^) ions. As opposed to iron based spinels, such as magnetite (Fe_3_O_4_), the divalent component is relatively inert with respect to the electrochemical series. Consequently, in redox reactions that may occur at the surface, the trivalent species are difficult to stabilize, rendering the surface oxygen species highly reactive. This enhanced reactivity can be observed in the selective oxidation of 2-propanol over Co_3_O_4_, in which a low temperature channel exists, if the surface of the precursor has been sufficiently oxidized prior to the catalytic reaction.^31^ In order to understand the impact of different gas environments on the reactivity, we titrated Co_3_O_4_ nanoparticles after exposure to reductive and oxidative atmospheres with CO in a separate setup (see Methods and Figure S4 in the Supporting Information).

**Figure 2:**
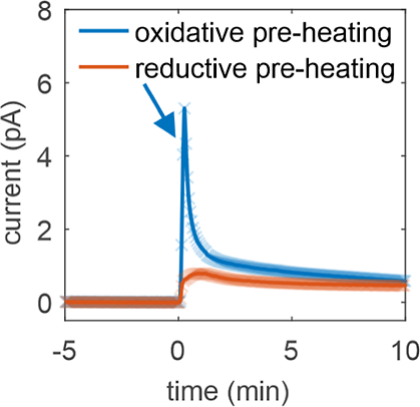
Surface titration using CO. CO_2_ formation at room temperature from the exposure of the Co_3_O_4_ spinel catalyst to 2% CO in He, indicated by the mass spectrometer currents for a mass/charge ratio of 44 after oxidative (10% O_2_ in He, blue curve) and mildly reductive (He, red curve) pretreatments at 250 °C for 1 h.

CO pulses have been applied at room temperature (RT) after heat treatment in oxidative (O_2_) and mildly reductive (He) environments at 250 °C (for titration experiments at 250 °C see Figure S5). Figure 2 shows the profiles of CO_2_ evolution, displayed as MS signals for a mass-to-charge ratio of 44, while exposing the nanoparticles to 2% CO in He after the two different pretreatments. There is a clear difference between the pretreatment in O_2_ versus He. While only little CO_2_ has been formed for the sample treated in He (red curve), a strong peak is observed in the CO_2_ signal corresponding to the sample treated in O_2_ (blue curve, blue arrow). Pretreatment in O_2_ seems to form active oxygenated species, which are highly active and capable of reacting with CO even at room temperature indicated by a sharp spike in the amount of CO_2_ at the reactor outlet. The blue curve in Figure 2 also implies that we deal with a stoichiometric reaction rather than a catalytic event. The drop of the CO_2_ signal over 10 min of CO exposure indicates that the active surface oxygen species are not fully replenished by the underlying bulk. Bulk oxygen diffusion to the partially reduced surface has been reported previously for Au/ZnO during CO titration experiments although using gas pulses in TAP (temporal analysis of products) studies and at higher temperatures above 240 °C.^32,33^ Nevertheless, we also performed an extended experiment, and we observed that the low intensity CO_2_ signal falls to a constant baseline. This suggests that underlying bulk oxygen can still slowly diffuse to the surface even at 20 °C but at a very limited rate. Note that care has been taken to completely remove the gas atmosphere after the different thermal treatments and prior to CO addition. Thus, no gas phase oxygen has been present in the reaction cell and the CO_2_ signal stems from the reaction of CO with surface oxygen. This finding suggests that electrophilic surface oxygen has been formed after the oxygen treatment, which is needed for CO_2_ formation using CO as a feedstock.

Low temperature CO oxidation, i.e. at RT or below, over Co_3_O_4_ catalysts has been demonstrated in various reports.^34−38^ The occurrence of this low temperature regime is generally attributed to a thermal activation in oxygen atmosphere. During the activation phase reactive oxygen species (ROS) at the surface are formed which are reacted off during CO oxidation causing the rapid deactivation of the catalyst. These ROS have been identified as superoxide species, which is a form of electrophilic oxygen.^39,40^ For electrochemical liquid phase oxygen evolution reaction (OER), the formation of such electrophilic surface oxygen for Co_3_O_4_ could be demonstrated as well.^41^ In addition it was shown that enhancing the amount of ROS improves the CO oxidation performance,^42^ while it was proposed that deactivation of this low temperature activity is due to reconstruction of the surface of the Co_3_O_4_ particles which hinders the regeneration of the catalyst.^43^

Co–O systems have been investigated intensively, where only CoO and Co_3_O_4_ are found to be thermodynamically stable phases^44^ at finite temperatures, while Co_2_O_3_ is only stable at low temperatures and high oxygen partial pressure.^45^ The transformation of Co_3_O_4_ nanoparticles to CoO was demonstrated on the local scale by heating in vacuum (reductive environment), with sufficiently low oxygen partial pressure.^46^ Navrotsky et al. could show that for nanosized Co–O systems, the CoO phase is stable only in a considerably narrower range within the phase diagram with respect to temperature and oxygen partial pressure compared to bulk Co–O systems.^47^ By studying the global potential energy surface, Kong et al. showed that solid phase transition between wurtzite CoO (h-CoO) and rock salt CoO (c-CoO) follow a one-way reconstructive phase transition with high energy barrier, while c-CoO and Co_3_O_4_ can form a biphasic junction, which implies reversibility of c-CoO and Co_3_O_4_ transition under reduction/oxidation conditions.^44^

This proves that the surfaces of a Co_3_O_4_ catalyst are significantly affected by the pretreatment environments and can form different kind of CoO_*x*_ surface structures. It is therefore required to apply and develop advanced techniques with high spatial resolution to track such surface structure variations and to understand the surface reactivity of the Co_3_O_4_ nanoparticles.

### 2.3 Geometric Structure Analysis by Local Imaging (TEM)

**Figure 3:**
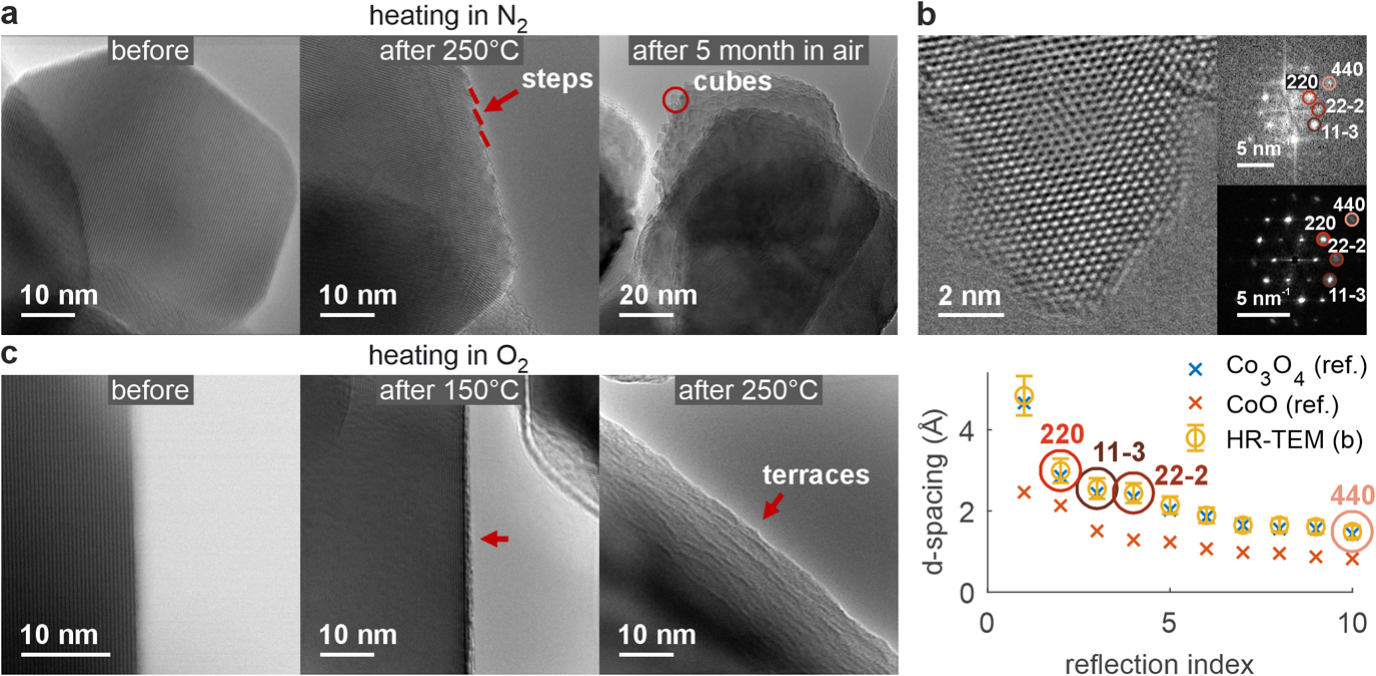
Structural changes as a function of thermal pretreatment (N_2_ vs O_2_ heating). (a) TEM images of a Co_3_O_4_ spinel nanoparticle before (left) and after heating in N_2_ (middle) and after another 5 months in air (right panel). (b) A magnified view of a cube-like structure (top left), the corresponding FFT image (top right) and an FFT image of a larger area of the same particle (center right). The circled diffraction spots in the FFT images are labeled with their corresponding Laue indices. The lattice spacings (d-spacing) of different planes of the particle are compared with reference data of Co_3_O_4_^48^ and CoO^49^ (bottom). (c) TEM images before (left), after heating in O_2_ at 150 °C (middle) and after further heating in O_2_ at 250 °C (right) (same particle, same surface).

Next, we will focus on the influence of different pretreatments on the structure of Co_3_O_4_ spinels. Figure 3 compares the different structural modifications of a Co_3_O_4_ nanoparticle heated under reductive (nitrogen) or oxidative (oxygen) conditions. Figure 3a shows TEM images of a Co_3_O_4_ spinel nanoparticle before and after heating in N_2_ at 250 °C using ILIAS in combination with quasi in situ electron microscopy and inert gas transfer. This allows us to track changes of the same particle and the same surface. The flat surface changed to a step-like surface as indicated by the red markers and the arrow in the middle panel of Figure 3a. After exposure of the same particle to ambient air for 5 months, cube-like structures have been formed on the surface (Figure 3a, red circle, right panel), indicating reoxidation of the particles to Co_3_O_4_ accompanied by further restructuring, which is corroborated by the local FFT analysis presented in Figure 3b. We derived the d-spacing values (Figure 3b, lower panel) via FFT analysis using CrystBox toolbox.^50^Figure 3c displays TEM images of another Co_3_O_4_ spinel nanoparticle before, after heating in O_2_ at 150 °C and after further heating in O_2_ at 250 °C (same particle, same surface). The initial flat surface (Figure 3c, left) became slightly roughened after heating in oxygen at 150 °C (Figure 3c, middle), and formed terrace-like structures at 250 °C (Figure 3c, right), as indicated by the red arrows in the middle and right panels.

These observations show that the thermal pretreatment has a major influence on the formation of the surface structure. Initially, the surface is flat and atomically sharp. After heating in different atmospheres at different temperatures, various types of restructuring take place as shown in Figure 3a,c (compare steps, cubes and terraces). Obviously, the order in which the treatment is applied plays an important role: While for a fresh surface heated in O_2_ the surface structure is slightly roughened and forms terraces (Figure 3c), a previously reduced sample that is then oxidized undergoes a more pronounced restructuring. This can be seen in Figures 1c and 3b (right images). In both cases, the sample was previously in a reduced state compared to Co_3_O_4_, i.e. CoO (Figure 1) and CoO_*x*_ (Figure 3). After oxidizing in O_2_ at 250 °C or in air at RT the sample forms cubic structures. Local TEM analysis revealed the crystal structure of these cubes: According to FFT analysis and comparison with literature values for Co_3_O_4_ and CoO (Figure 3b), we attribute the cube-like particles to a Co_3_O_4_ spinel structure. The d-spacings derived from the FFT pattern agree within measurement accuracy with the Co_3_O_4_ reference (yellow circles and blue crosses), while the CoO reference (red crosses) differs significantly. To highlight the correlation between the FFT diffraction spots and their corresponding d-spacings, four diffraction spots are circled and are labeled with their Laue indices.

Although we could derive the crystallographic phase (Co_3_O_4_ spinel) of the cube-like structures on the surface of the catalyst particles (for their size distribution see Figure S6 in the Supporting Information), a detailed understanding of the step and terrace-like structures in terms of their crystallographic phase that form under different conditions remains challenging. There are several reasons for this: first, and most importantly, the transition from Co_3_O_4_ to CoO in terms of oxidation state is partially continuous as will be discussed below, which also affects the lattice spacing and prevents a clear assignment of the surface state by imaging alone. Second, the inhomogeneity of the sample in terms of size and shape hinders clear interpretation of all images (the larger the particles, the less likely it is to obtain high-resolution information). Third, the sample must be tilted exactly to the zone axis, which was possible to a limited extent only using a vacuum transfer single-tilt holder (see Methods). For these reasons, we complement imaging (STEM) of the catalyst with simultaneously acquired spectroscopy (EELS) to access the electronic structure (see Methods section for details). In this way, we are able to follow the oxidation state across the surface of a Co_3_O_4_ spinel nanoparticle—from vacuum to surface to bulk—with subnanometer resolution. By mapping the electronic structure via high resolution ELNES measurements, we will lay the groundwork to address the question of surface oxygen species experimentally.

### 2.4 Electronic Structure Analysis by Local Spectroscopy (EELS)

**Figure 4:**
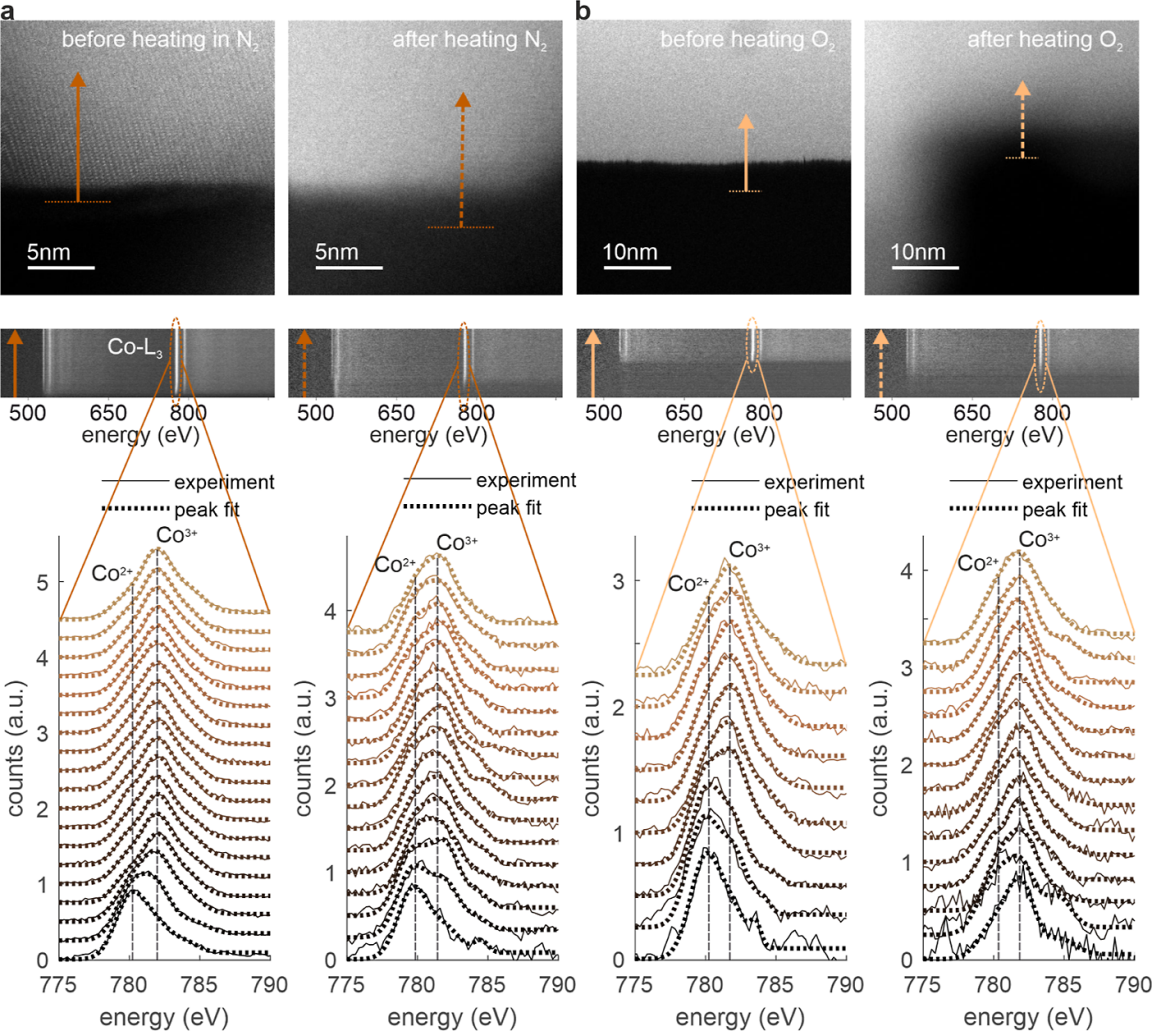
Spectral evolution across the surface (EELS): (a) HR-STEM images of the same surface of a Co_3_O_4_ spinel nanoparticle before and after heating in N_2_ (top), 2D views of the EEL line scans along the arrows in the HR-STEM images (middle), corresponding EELS plots of the Co L_3_-edge (bottom), extracted from the region indicated by the ellipses in the middle panel. The experimental spectra (solid lines) are compared with the peak fits (dotted line). The positions of the Co^2+^ and Co^3+^ subpeaks are marked by vertical dashed lines. (b) Same as (a), but for a different sample heated in O_2_ instead of N_2_. The arrows indicate the position and direction of the EEL line scans. The length of the line scans is 10 nm.

Access to the spectral properties of the surface of the cobalt oxides as a function of pretreatment requires the development of dedicated hardware, but also the development of suitable software to track the oxidation state across the surface on the same particle before and after heating. The former has already been introduced with the quasi in situ setup and is described in the Supporting Information (Figure S1). The latter is applied in Figure 4, where the spectral evolution across the surface before and after heating in N_2_ or O_2_ atmospheres is presented. For interpreting the EEL spectra, we focus on the relative contribution of two subpeaks to the Co L_3_ peak. We will demonstrate how the position dependent oxidation state can be approximated from this subpeak ratio. We would like to note that the O K fine structure could, in principle, also be used to get information on the local oxidation state and the electrophilic and nucleophilic character of oxygen. However, the limited signal-to-noise ratio of the O K fine structure prevented a clear interpretation. This is why we focus on the Co L_3_ white line, that gives a better signal-to-noise ratio. Figure 4a shows HR-STEM images of the identical surface of a Co_3_O_4_ spinel nanoparticle before (top left) and after (top right) heating in N_2_. The corresponding 2D views of the EEL line scans before (middle, left) and after heating in N_2_ (middle, right) are shown below. The horizontal direction is associated with the energy loss, while the vertical direction of the 2D maps is related to the line scan position and the intensity to the EEL probability. The waterfall plots below depict the Co L_3_-edge before (lower left) and after (lower right) heating in N_2_. The spectral evolution across the interface is extracted from the lateral positions as indicated by the arrows in the top panel and from the spectral energy range as indicated by the ellipses in the middle panel. The experimental spectra (solid lines) are compared with the peak fits (dotted line). Subpeak features are highlighted by vertical dashed lines, referring to the Co^2+^ and Co^3+^ contributions to the Co L_3_-peak. In Figure 4b we follow the same approach but the sample has been heated in O_2_ instead of N_2_, also at 250 °C. At this point we would like to emphasize the peak-fitting routine developed for this purpose, which is described in more detail in the Methods section. The results of the fitting routine are corroborated by an independent approach based on the linear combination of experimental reference spectra for Co^2+^ (CoO) and Co^3+^ (LaCoO_3_) measured on the same microscope under the same conditions. We compare the results of both approaches in the Supporting Information in Figures S7 and S8.

**Figure 5:**
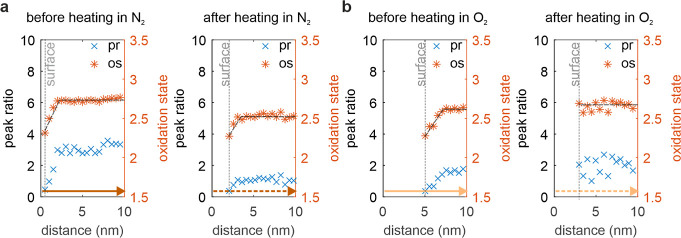
Evolution of the oxidation state across the surface (EELS). (a) Peak ratios (pr) and oxidation states (os) before and after heating in N_2_. The vertical dotted lines indicate the surface position of the nanoparticle, the dashed lines are a guide to the eye. (b) Peak ratios (pr) and oxidation states (os) before and after heating in O_2_. The arrows indicate the direction of the line scans (see also the arrows in Figure 4).

A closer look at the waterfall plots reveals an increased Co^2+^ contribution after N_2_ heating (Figure 4a, bottom right) toward the subsurface and bulk compared to the spectra from this region before heating (Figure 4a, bottom left). In contrast, the Co^2+^ contribution after O_2_ heating (Figure 4b, bottom right) is lower at the surface compared to the slightly reduced (i.e., higher Co^2+^ peak) surface before heating (bottom left). From these results we conclude that variations in the oxidation state that depend on the relative contribution of the two Co L_3_ subpeaks can be tracked. Assuming a (maximum) oxidation state of 3 with contribution only from the Co^3+^ subpeak and a (minimum) oxidation state of 2 with contribution only from the Co^2+^ subpeak, we derived an oxidation state analogon os from the Co L_3_ subpeaks (Co^2+^ and Co^3+^) given by
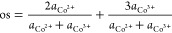
1where  and  are the amplitudes of the two associated Gaussian fits (see Methods). This allows us to follow the oxidation state across the interface (Figure 5). Figure 5a shows the evolution before (left plot) and after heating in N_2_ (right plot), as obtained from the data shown in Figure 4a. The peak ratios (blue crosses, pr) are plotted together with the oxidation states (red crosses, os) derived from (eq 1) and based on the Co^2+^ to Co^3+^ peak ratio using our peak fitting approach (Methods and Figure S9 in the Supporting Information). The surface position is indicated by a vertical dotted line, while the dashed lines are a guide for the eye to follow the evolution of the oxidation state as a function of its position and pretreatment. It is important to note that in addition to the surface, which remains more reduced compared to the bulk region, the subsurface and bulk regions are at overall lower values after heating compared to their initial state. Figure 5b shows the evolution of peak ratios and oxidation states before (left plot) and after heating in O_2_ (right plot), derived from the data shown in Figure 4b. In contrast to the heating experiment in N_2_, the surface after heating in O_2_ is almost at the same level as the bulk, as can be seen by the flattened gray line, and there is no drop in oxidation state toward the surface. These results demonstrate that we are able to simultaneously track the oxidation state together with high spatial imaging at a location relevant for the catalytic reaction, i.e. the surface of the catalyst. We would like to emphasize that existing approaches are often not sufficient to extract these small variations in the oxidation state, as shown in the Supporting Information in Figure S10.

### 2.5 Electronic Structure Analysis by X-ray Techniques (NAP-XPS and TXM)

**Figure 6:**
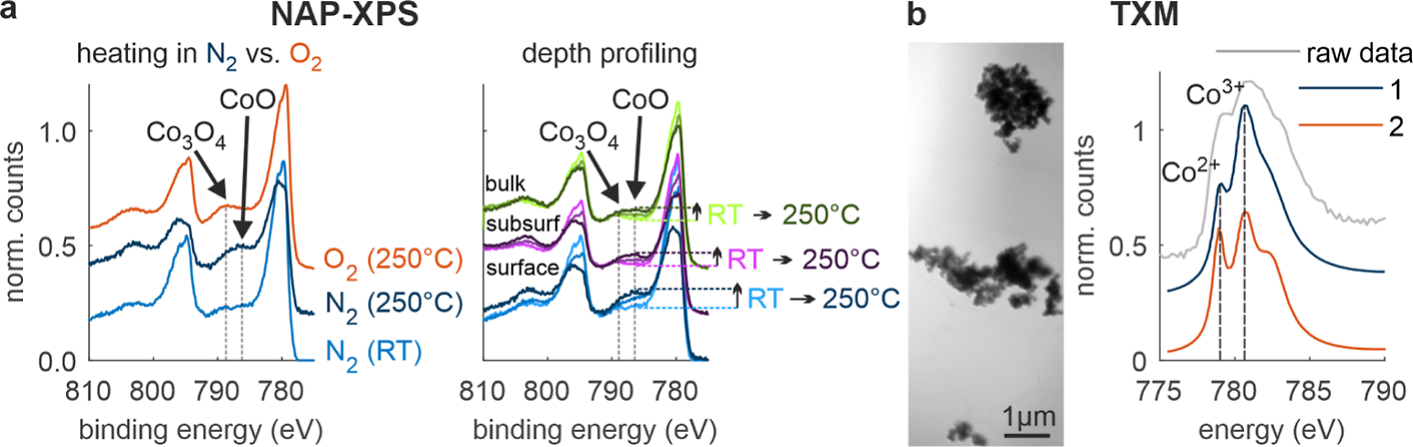
(a) Heating and depth profiling by X-ray spectroscopy (NAP-XPS). Surface sensitive NAP-XPS spectra of the Co-2p edges acquired in O_2_ at 250 °C (red), in N_2_ at 250 °C (dark blue) and in in N_2_ at room temperature (blue). Depth profiling and heating in N_2_. Co-2p edges with surface, subsurface and bulk sensitivity for different temperatures, as indicated by the labels on the right side (see text). (b) TXM before heating. TXM image (left) and TXM spectrum (right, gray), decomposed in component 1 (blue) and component 2 (red).

To corroborate the EELS results, we performed NAP-XPS using similar protocols (Methods). The sample was measured either in N_2_ or O_2_ at RT, 100 and 250 °C and XPS spectra were simultaneously acquired (Figure 6, for XPS quantification results see Figure S11 in the Supporting Information). Figure 6a, left, shows surface sensitive NAP-XPS spectra (*E*_kin_ = 150 eV) of the Co-2p region (corresponding to Co L_3,2_) acquired in O_2_ at 250 °C (red line), in N_2_ at 250 °C (dark blue line) and in N_2_ at room temperature (blue line). The spectrum features satellites, highlighted by the vertical dashed lines, that act as finger prints for the oxidation state. These satellites can be attributed to Co_3_O_4_ (left, higher binding energy) and CoO (right, lower binding energy).^51^ While for heating in O_2_ the satellite maximum shifts to the left (black arrow pointing to the red line), for heating in N_2_ this maximum shifts to the right (black arrow pointing to the dark blue line). Interestingly, for the sample at RT, both components are slightly visible (compare blue with red and dark blue curves), indicating a slightly reduced surface even before heating and in agreement with the EELS results (Figure 5). In Figure 6a, right, the sample was heated from RT via 150 to 250 °C in N_2_. From the surface (*E*_kin_ = 150 eV, blue lines) to the subsurface (*E*_kin_ = 300 eV, purple lines) to the bulk (*E*_kin_ = 600 eV, green lines, see also the Methods section for signal depths), the XPS spectra show a decreasing CoO component at RT (follow the right dashed vertical line, labeled CoO). At higher temperatures (100 °C and even more so at 250 °C) the increased CoO component, even in the bulk, confirms the (partial) reduction of the Co_3_O_4_ spinel nanoparticles, which we also found by EELS, as shown in Figure 5a.

Figure 6b complements the study by transmission X-ray microscopy (TXM). Similar to STEM-EELS, TXM combines microscopy with spectroscopy. The left panel shows a TXM image of the same powder sample as used for the EELS and NAP-XPS investigations before heating. The contrast is formed by integrating 150 frames acquired at X-ray energies ranging from 775 to 805 eV in 0.2 eV steps (see Methods). The right panel displays a TXM spectrum integrated over all image pixels (gray), together with the two principal components (1, blue and 2, red). These components were obtained by decomposing the raw data using a multiparameter gradient descent approach, i.e. a reference free data analysis method that is advantageous compared to conventional principal component analysis (PCA) or non-negative matrix approximation (NNMA). Although the spatial allocation of the two components could not be resolved, they confirm the two phases, that are the spinel phase (blue) and a reduced phase (red)—with an increased Co^2+^ contribution—in accordance with the findings by EELS and XPS. For the sake of completeness, we would like to mention that beside fast electrons or X-rays used as a local probe, also other scanning probe techniques, such as scanning electrochemical probe microscopy (SEPM) or scanning tunneling microscopy (STM) can be applied in a wide range of operando setups.^52,53^

### 2.6 Theory and Simulation (DFT)

**Figure 7:**
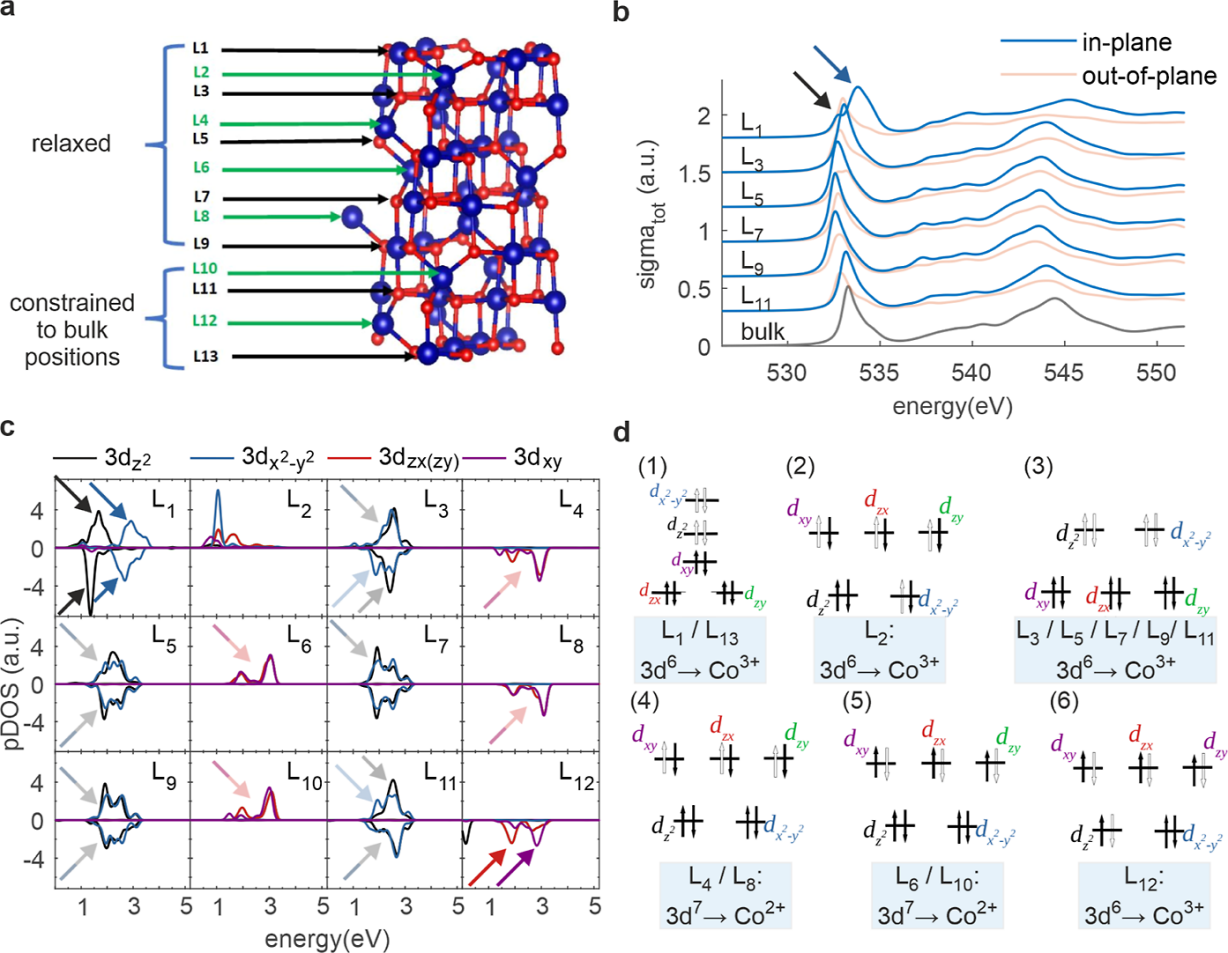
DFT simulations. (a) Co_3_O_4_ spinel structure with B-terminated surface (001). Blue and red spheres represent Co and O atoms, respectively. (b) O K-edge XANES spectra of oxygen-containing layers compared to the bulk (black line). (c) pDOS on selected cobalt atoms (d-bands), each representing all cobalt within the corresponding layer. The arrows highlight the peaks above the Fermi energy (*E* = 0 eV, unoccupied states) that potentially contribute to the pre-edge peak in the O K-edge spectra. (d) Crystal field splitting diagrams (CFSD) in 3d shells of cobalt atoms. The occupation scheme (black arrows) causes the oxidation state as indicated by the blue boxes.

Finally, we will discuss the electronic structure of Co_3_O_4_ at and near the surface by DFT simulations as shown in Figure 7. The considered structure has a B-terminated surface (001), i.e. a 001 surface of a spinel structure, consisting of square pyramidal coordinated Co atoms and O atoms, with relaxed atomic layers L_1_–L_9_ on top of layers L_10_–L_13_ which are constrained to bulk positions (Figure 7a). The blue and red spheres represent Co and O atoms, respectively. L_2*n*–1_ are oxygen-containing layers, while layers L_2*n*_ contain no oxygen, where n is a natural number. In Figure 7b the O K-edge XANES spectra of the layers L_2*n*–1_ are compared with the one calculated in the bulk Co_3_O_4_ (black line, energy is shifted by 531.45 eV according to the experimental O K energy value). The XANES spectra are computed using XSpectra code included in Quantum-Espresso package, with the same parameters as described in our recent works on the O K-edge in Co_3_O_4_ systems (see Methods). While the blue spectra represent the in-plane contribution, the red spectra are related to the out-of-plane components of the cross section. As the in-plane component is more relevant for comparison with EELS (the electron beam points in the in-plane direction), the blue lines are more pronounced than the red lines. The O K (i.e., O-1s) pre-edge peak is split into two peaks and in the case of layer L_1_ (black and blue arrow). Figure 7c shows the projected densities of states (pDOS) on cobalt atoms (Co-3d bands) belonging to oxygen-containing layers L_2*n*–1_ and oxygen-free layers L_2*n*_. Lines with positive values correspond to spin-up, lines with negative values to spin-down DOS components. Only peaks above the Fermi energy (*E* = 0 eV) up to *E* = 5 eV are shown (unoccupied states). This is the range that is relevant to the discussion of the O-1s prepeak due to its origin in the hybridization of Co-3d and O-2p states.^54^ The full energy range of the pDOS, including occupied states, is shown in the Supporting Information in Figure S12. The arrows highlight peaks from the different d-bands, whether overlapping at the same energy (single arrow) or separated at two different energies (two arrows). Figure 7d displays crystal field splitting diagrams (CFSD) within the 3d shells of the cobalt atoms. The black filled arrows represent occupied electron states, while the hollow arrows characterize empty electron states. (1) Square pyramidal CFSD of Co^3+^ ions with electron configuration *e*^4^*b*_2_^2^*b*_1_^0^*a*_1_^0^ for the layers L_1_ and L_13_. (2) Tetrahedral CFSD of Co^3+^ ions with electron configuration *e*^3^*t*_2_^3^ for the layer L_2_. (3) Octahedral CFSD of Co^3+^ ions with electron configuration *t*_2g_^6^*e*_g_^0^ for the layers L_3_, L_5_, L_7_, L_9_ and L_11_. (4) Tetrahedral CFSD of Co^2+^ ions with electron configuration *e*^4^*t*_2_^3^ for the layers L_4_ and L_8_. (5) Tetrahedral CFSD of Co^2+^ ions with electron configuration *e*^4^*t*_2_^3^ for the layers L_6_ and L_10_. (6) Tetrahedral CFSD of Co^3+^ ions with electron configuration *e*^3^*t*_2_^3^ for the layer L_12_. From these diagrams the electron configuration and therefore the oxidation state can be extracted, which is shown in the blue boxes below.

From this simulation study we conclude that the spectral shape of the O K-edge at and near the surface is different from the bulk response of a Co_3_O_4_ spinel. In particular, the pre-edge peak splits into two subpeaks at the surface atomic layer (L_1_), which we interpret as a consequence of a splitting of the Co-3d_*z*_^_2_^ and Co- pDOS in the same layer (compare black and blue arrows in Figure 7b,c). This is because the origin of the prepeak is attributed to the hybridization of the O-2p with the Co-3d states. Since the influence of the surface on the oxygen-containing layers L_3_–L_11_ is smaller, no splitting can be observed, but tiny energy shifts in the prepeak. Again this goes hand in hand with the shape of the Co-3d_*z*_^2^ and Co- pDOS (no splitting but tiny variations/shifts between layers). In all oxygen-containing layers L_2*n*–1_ Co is octahedrally coordinated with oxidation state 3 (except L_1_), alternating with oxygen-free layers L_2*n*_ where Co is tetrahedrally coordinated with oxidation state 2 (except L_2_). Interestingly, L_1_, which is in square pyramidal coordination instead of octahedral, influences the oxidation state of the tetrahedrally coordinated L_2_, which becomes 3 instead of 2. This is a clear indication that the surface has a direct influence on the oxidation state of the near-surface layers of the spinel structure. Furthermore, the tiny energy prepeak shifts of successive spectra are more noticeable as one moves away from the layers that are constrained to the bulk positions toward the relaxed atomic layers. This may indicate the increase in the atomic distortions.

If we compare the simulation results with our experimental findings, the simulations most closely reflect the situation of a surface mimicking Co_3_O_4_ (see Figure 5b, right), since the interaction of the surface with air or pure nitrogen was not taken into account in the simulations. From the simulation results we would expect an alternating oxidation state between individual atomic layers. Indeed, we observed periodic variations of the oxidation state for 0.5 nm steps (see Figure S13). This is a surprising result, as neither the visual impression of the raw spectral data nor the slight mismatch of the zone axis condition would suggest this finding. However, both the peak fit approach and the linear fit approach show the same trend, implying that we have uncovered atomic scale oxidation state variations in agreement with the DFT simulation results.

Note, a direct comparison of simulated and experimental spectra for the O K- or Co L_3,2_-edge has not yet been possible for the following reasons: to date, the simulation of Co L_3,2_ XANES spectra by DFT-based single-electron approaches for a system as described in this paper is not straightforward. The O K-edge is much better suited to study the electronic structure of this system by DFT. On the other hand, the experiment lacks a sufficient signal-to-noise ratio for the O K-edge, which hinders a clear interpretation of the ELNES. In addition, the relationship of the O K ELNES to the oxidation state is more complex and ambiguous. Therefore, the Co L_3,2_-edge was used instead in the experiment. For future work, we believe that a direct comparison of experimental and simulated spectra, whether the O K or the Co L_3,2_-edge, might be beneficial.

## 3 Conclusion

We introduced ILIAS in combination with quasi in situ electron microscopy as an alternative or supplementary technique to operando electron microscopy. As a proof of concept, we applied ILIAS on Co_3_O_4_ spinel nanoparticles and demonstrated how this approach enabled the tracking of the oxidation state with subnanometer resolution before and after oxidative or reductive heating on identical Co_3_O_4_ nanoparticle surfaces. The geometric structure (imaging) and its restructuring due to different gas treatments was simultaneously measured with variations in the electronic structure (spectroscopy). Subsequent CO titration showed a strong dependence of the surface reactivity on its thermal pretreatment to form CO_2_. Preheating the catalyst in a reductive environment led to a continuous, but low CO_2_ production, whereas preheating in an oxidative environment resulted in a remarkable CO_2_ peak even at room temperature. We could relate this different behavior to the observation of different surface restructuring—from flat to stepped and from flat to terraced surface structures, respectively. In the case of a reduced and subsequently oxidized sample, an even more pronounced restructuring was observed in the form of cube-like structure. Further insight was gained by extending the consideration of geometric structure to the elucidation of the electronic structure of the identical particle surface. This has been achieved by developing hardware (ILIAS in combination with a quasi in situ setup) and software (peak fitting vs linear combination) for the evaluation of the ELNES of Co-L_3,2_ edges to track the oxidation state across the surface of the catalytic nanoparticle. We observed a variation of the oxidation state from surface to subsurface to bulk as a function of pretreatment with subnanometer resolution. The EELS results were confirmed by additional X-ray spectroscopy (NAP-XPS and TXM). Furthermore, we were able to show a periodic change in the oxidation state with a period of about 5 Å for a Co_3_O_4_ spinel nanoparticle, in agreement with DFT simulations. ILIAS in combination with quasi in situ electron microscopy therefore opens up a promising way of approaching the electronic and geometric structure of a catalyst toward atomic resolution with better signal-to-noise ratio, less beam impact and higher pressure and temperature capabilities compared to operando approaches.

## 4 Methods

### 4.1 Experimental Methods

#### 4.1.1 Quasi In Situ Setup

The quasi in situ setup developed for this work corresponds to a completely revised concept based on a solution proposed by Masliuk et al. for the use of a TEM microreactor described in ref (55). The flowchart of this setup is shown in the Supporting Information in Figure S1, with the corresponding components labeled in Figure S2. Multiple inlet gas lines, laser heating up to 500 °C, a high pressure range up to 30 bar, and a mass spectrometer are the main features of the setup. Six inlet gas lines can be combined for gas mixing, each with an independent mass flow controller. In addition, inlet gases or mass flow controllers can be easily changed for specific purposes, making this facility a highly flexible tool. The system is enclosed in a housing and connected to an exhaust pipe, which provides a constant volume exchange by extracting the internal volume of the system. This allows the use of harmful gases. The laser power is 40 W, operating at a wavelength of 808 nm (LIMO40-F200-DL808-LM, Lissotschenko Mikrooptik). Laser heating is PID-controlled to raise and maintain the temperature as required. The quasi in situ reactor can be easily connected or replaced by other TEM grid reactors^56^ (dedicated for different purposes) via two quick connectors for inlet and outlet. The outlet is connected to a valve that allows switching between a proton transfer reaction mass spectrometers or directly to the exhaust line. The reactor itself contains the TEM grid with the sample previously analyzed in the TEM and transferred to the reactor. After heat and gas treatment in the quasi in situ setup, the sample in the reactor is transferred back into a TEM vacuum transfer holder (VTST 4006, Ametek) in a glovebox and analyzed again in the TEM using ILIAS.

The heating experiments for quasi in situ investigations (Figures 1, 3–5 and S7–S10) have been performed at 5 N mL/min gas flow (N_2_ or O_2_) at 2.3 bar up to 150 or 250 °C with a heating ramp of 2 °C min^–1^. After the heating, the microreactor was filled with argon at 2.8 bar before the sample was transferred to the vacuum transfer TEM holder inside a glovebox.

#### 4.1.2 X-ray Diffraction

The X-ray diffraction (XRD) measurement (Figure S3) was performed in Bragg–Brentano geometry on a Bruker AXS D8 advance II theta/theta diffractometer, using Ni filtered Cu Kα_1+2_ radiation and a position sensitive energy dispersive LynxEye silicon strip detector. The sample powder was filled into the cavity of a low background Si single crystal sample holder, the surface of the powder bed being level with the sample holder edge (front loading). The diffraction pattern was recorded in continuous scanning mode in the range of 6–140° 2θ with an increment of 0.02° and a counting time of 1 s/step, resulting in a total accumulation time of 185 s per data point. Phase identification was performed using DIFFRAC. SUITE EVA (Bruker AXS, 2010-2018) in combination with the PDF-4+ database (ICDD, 2018). XRD data were then evaluated by whole powder pattern fitting according to the Rietveld method as implemented in the TOPAS software (version 5, Bruker AXS, 1999-2014). The crystal structure used for the Rietveld fits was obtained from the ICSD Web database (FIZ Karlsruhe).

#### 4.1.3 Thermogravimetric Mass Spectrometry

Thermogravimetric mass spectrometry (Figure S4) was accomplished on a NETZSCH STA 449C Jupiter instrument equipped with an electromagnetic microbalance with top loading. The instrument was coupled via quartz capillary (heated to 393 K) to a Pfeiffer OmniStar quadrupole mass spectrometer for gas-phase analysis. Approximately 20 mg of the catalyst sample was placed into a corundum crucible (85 μL) without lid and heated to 1073 K with a heating ramp of 2 K min^–1^. The used gas atmosphere was 99.999% Ar with a total gas flow of 100 N mL/min. The resolution of the microbalance was 0.1 μg and the relative error of mass determination 0.5% The temperature was calibrated using conventional pure metal standards: In, Sn, Bi, Zn, Al, Ag and Au. The MS experiments were performed using electron ionization energy of 70 eV and a dwell time per mass of 0.2 s.

#### 4.1.4 Electron Microscopy and Electron Spectroscopy

TEM, STEM and EELS measurements were performed using a probe and image corrected JEOL JEM-ARM200F transmission electron microscope operated at 200 kV. For STEM-EELS experiments, a semi-convergence angle (alpha) of 15.6 mrad and a semi-collection angle (beta) of 20.1 mrad and a dispersion of 0.25 eV/channel were chosen. The beam current was 30 pA using a cold FEG as the electron source. Dual EELS was used to correct for energy drift and to deconvolve the high loss spectrum using the Fourier ratio method. The point resolution of the microscopes was in the range of or below 100 pm. To verify the resolution capabilities of the microscope, Si dumbbells on a Si(110) test sample could be clearly resolved by imaging (HAADF and BF) and a simultaneously measured EDX line profile (Figure S14). The crystal structure and orientation analysis in Figure 3 was performed using CrysTBox Toolbox.^57^ To achieve a sufficient signal-to-noise ratio in the case of EEL measurements, EEL line scans of 200 pixels and a total length of 10 nm were averaged by 10 neighboring pixels, resulting in a final step size of 0.5 nm between the EEL spectra shown in Figures 4 and 5 and S7–S10. In addition, the beam was scanned in perpendicular direction to the line scan direction over a distance of 5 nm to spread the dose and reduce beam damage (dose averaging, see horizontal, dotted lines in the TEM micrographs in Figure 4a,b).

#### 4.1.5 Peak-fitting Routine

The oxidation state values discussed in Figure 5 have been extracted from the EEL spectra shown in Figure 4 by applying a peak-fitting routine demonstrated in the Supporting Information in Figures S7 and S8. The results were then compared with an independent approach based on a linear combination of experimental EEL spectra of CoO (Co^2+^) and LaCoO_3_ (Co^3+^) measured on the same microscope under the same conditions to confirm the robustness and significance of the fit [Figure S8 (Movie S1)]. The fit approach is based on a Matlab script that uses internal Matlab functions to enable the overall fit. First, the experimental spectra were energy drift corrected and deconvolved by the Fourier ratio method using the corresponding low-loss spectra. Next, the residual oxygen signal was subtracted by fitting a power law function in front of the Co L_3,2_-edges using a fit range from 700 to 770 eV. The Co signal in the range from 770 to 805 eV was then fitted by a function consisting of 6 Gaussians and two step functions (normal cumulative distribution function, normcdf). Several constraints were imposed to reduce the total number of fitting parameters: These were a fixed 2:1 ratio for the two step functions modeling the Co L_3_- and Co L_2_-edge with respect to their step height. The position, width and edge energy difference were set to 781.9, 1 and 14.6 eV respectively, in accordance with reference data and instrumental energy broadening. On top of the edge step functions, 3 Gaussians for the Co L_3_ white line and 3 Gaussians for the Co L_2_ white line were fitted simultaneously, with the allowed energy values restricted to either Co L_3_ (775 to 790 eV) or Co L_2_ (790 to 805 eV). To avoid a divergence of the peak widths, the allowed Gaussian peak widths were chosen in the range from 0.5 to 1.5 eV. Finally, the energy of the second Gaussian (Co^3+^ contribution) was constrained to a fixed energy value of 781.9 eV, i.e. the position of the underlying edge step function. For the linear combination approach, the linear factors α and β were found by minimizing the difference between the experimental spectra and the linear combination (α·*y*_CoO_+β·*y*_LaCoO_3__). *y*_CoO_ and  are the experimental spectra of the reference materials CoO (Co^2+^) and LaCoO_3_ (Co^3+^), respectively, measured under the same conditions on the same microscope as the cobalt spinel sample. The difference was minimized using a Matlab internal function (fmincon).

#### 4.1.6 X-ray Spectroscopy (NAP-XPS)

Synchrotron-based near-ambient pressure X-ray photoelectron spectroscopy (NAP-XPS)measurements were conducted at the CAT end station using the soft X-ray branch of the EMIL beamline (UE48 PGM) at BESSY II.^58^ The station was equipped with a differentially pumped Specs Phoibos 150 NAP hemispherical sector analyzer, coupled to a 2D delay line detector, which was set to detect photoelectrons with a pass energy of 10 eV.

For the measurements, the chamber (with a base pressure in the high 1 × 10^–8^ mbar range) was backfilled to 0.5 mbar with either N_2_ or O_2_. The sample was pressed to a pellet (8 mm diameter), which was mounted onto a sapphire sample carrier and attached to a type K thermocouple on the spectrometer-facing side of the pellet. Heating was provided by an infrared laser from the rear. In order to obtain a depth profile, the spectra were recorded at different kinetic energies of 150, 300 and 600 eV. This corresponds to inelastic mean-free paths of 0.6, 0.8 and 1.3 nm in Co_3_O_4_ according to the TPP-2 M equation.^59,60^ The acquired spectra were energy-corrected using the valence band spectra, which was cross-checked with C 1s spectra (adventitious carbon).

#### 4.1.7 X-ray Microscopy (TXM)

Measurements were performed at the U41-PGM1-XM beamline at the BESSY II electron storage ring.^61^ The NEXAFS spectra were collected by using a charge coupled detector (CCD) of 1340 pixel × 1300 pixel with each pixel corresponding to 10 nm sampling size. The used Fresnel zone plate objective had an outermost zone width of 25 nm corresponding to a lateral resolution of approximately 30 nm. The energy sampling was 0.2 eV in the photon energy range from 775 to 805 eV while the spectral resolution with a monochromator slit width of 15 μm is better than 150 meV. The exposure time per image was 6 s.

From the acquired spatially resolved energy stack a region of interest was identified using a multiparameter gradient decent approach which also estimates the background and the mean intensity without sample by fitting beers law to the acquired data. Finally the mean value of the logarithm of the quotient between acquired intensity and mean intensity without sample was used to generate the raw data for Figure 6b.

#### 4.1.8 Surface Titration Using CO

40 mg of the Co_3_O_4_ catalyst fine powder (Sigma-Aldrich, product number 637025, calcined at 600 °C for 3 h) was loaded into a microreactor and pretreated for 1 h at 250 °C in either mildly reductive atmosphere i.e. He or oxidative i.e. 10% O_2_ in He. The flow rate passing through the catalyst was set to 80 N mL/min. After cooling down to RT in both cases, the sample was exposed to a 2% CO in He while the mass to charge ratio of 44, indicative of CO_2_, was monitored using a mass spectrometer (Omnistar GSD 320, Pfeiffer Vacuum, Wetzlar, Germany). The choice of He instead of N_2_ was due to overlapping of N_2_ and CO molecular masses.

### 4.2 Computational Methods

The calculations have been performed using the Quantum-ESPRESSO (QE) software package^62^ based on the density functional theory (DFT) in the framework of pseudopotential approach using the plane wave method. We used the ultrasoft pseudopotential (USPP)^63^ to describe the interactions between the ion cores and valence electrons, while the generalized gradient approximation (GGA) together with a *U* (Hubbard correction) have been used to describe the electron–electron interactions. A *U* value of 3.5 eV was used as it was found sufficient to reproduce the correct band gap as well as structural and magnetic properties of Co_3_O_4_, in good agreement with the experimental data.^64,65^ Furthermore, a plane wave cutoff energy of 40 Ry and a 6 × 6 × 6 *k*-point mesh sampling of the Brillouin zone were necessary.

The XANES spectra have been calculated using the XSPECTRA code^66^ which is a module in the QE software package. Therein, the X-ray absorption cross-section is expressed in terms of a transition operator coupling the initial and the final states which are solutions of the Kohn–Sham (KS) equations. In the case of the K-edge, the initial state is a core 1s orbital obtained from an isolated absorbing atom in the absence of a core hole, while the final state is obtained self-consistently through the resolution of the KS equations for the whole system, including core hole effects in the pseudopotential of the absorbing atom.

The mathematical basis of X-ray absorption spectroscopy in the USPP scheme and the use of the PAW method to reconstruct the all-electron wave function, and to obtain the XANES intensities have been described in ref (66). In practice, the electric dipole cross-section is calculated for a given polarization direction using the Lanczos algorithm and the continued fraction method.^67^ This approach does not require an explicit calculation of empty states and is very fast, since only the charge density is needed. It should be noted that generally, experimental X-ray absorption spectra contain fewer structures and display broader features than theoretical spectra. This is due to the fact that finite lifetime of the core-hole is usually neglected in the theoretical calculations.

To facilitate proper comparison between theory and experiment, the calculated spectrum is modified so that the finite core-hole lifetime is accounted for. A convenient way that is often employed to achieve this is to convolve the raw spectrum a posteriori with a Lorentzian.^68^ For this purpose, we used γ = 0.3 eV for the incident photon energy of up to 1.5 eV and γ = 0.8 eV above a photon energy of 10 eV while γ varies linearly in the intermediate photon range between the two photon energy values. We recently applied such a Lorentzian convolution to reproduce the experimental spectrum for the bulk Co_3_O_4_^64^ and to monitor the oxidation of 2-propanol on the Co_3_O_4_ surface.^69^

## Supporting Information Available

Quasi in situ setup, components of setup, X-ray diffractogram of Co_3_O_4_, TG-MS measurements of Co_3_O_4_, peak fit vs linear combination approach of localized EEL spectra, link to movie, oxidation state evolution across the surface, comparison with commercially available software, pDOS of Co d-states including occupied states, EEL spectra after heating in O_2_, resolution tests (PDF)
Movie of EEL spectra series across the surface of a Co_3_O_4_ nanoparticle (AVI)

## Supplementary Material

am4c20690_si_001.pdf

am4c20690_si_002.avi
