# Supplementary material for: Subnanometer Tracking of the Oxidation State on Co3O4 Nanoparticles by Identical Location Imaging and Spectroscopy
Source: ACS Appl Mater Interfaces. 2025 Jan 31;17(6):9419–30. doi: 10.1021/acsami.4c20690 (PMC11826872; doi:10.1021/acsami.4c20690)
Supplement: Supplementary file 1 — am4c20690_si_001.pdf [file am4c20690_si_001.pdf]

Supporting Information: Subnanometer Tracking of the Oxidation State on Co_3O_4 Nanoparticles by Identical Location Imaging and Spectroscopy

Franz-Philipp Schmidt,[*,†] Thomas Götsch,[†] Sharif Najafishirtari,[‡,¶] Malte Behrens,[‡,¶] Christoph Pratsch,[§] Stephane Kenmoe,[‖] Dick Hartmann Douma,[⊥] Frank Girgsdies,[†] Jasmin Allan,[†] Axel Knop-Gericke,[†,#] and Thomas Lunkenbein[*,†]

†*Department of Inorganic Chemistry, Fritz-Haber-Institut der Max-Planck-Gesellschaft, Germany*

‡*Institute of Inorganic Chemistry, Christian-Albrechts-Universität zu Kiel, Germany*

¶*Kiel Nano, Surface and Interface Science KiNSIS, Christian-Albrechts-Universität zu Kiel, Germany*

§*Department X-Ray Microscopy, Helmholtz-Zentrum Berlin für Materialien und Energie GmbH, Germany*

‖*Department of Theoretical Chemistry, University of Duisburg-Essen, Germany*

⊥*Faculté des Sciences et Techniques, Groupe de Simulations Numériques en Magnétisme et Catalyse, Université Marien Ngouabi, Congo*

#*Department of Heterogeneous Reactions, Max Planck Institute for Chemical Energy Conversion, Germany*

E-mail: schmidt@fhi-berlin.mpg.de; lunkenbein@fhi-berlin.mpg.de

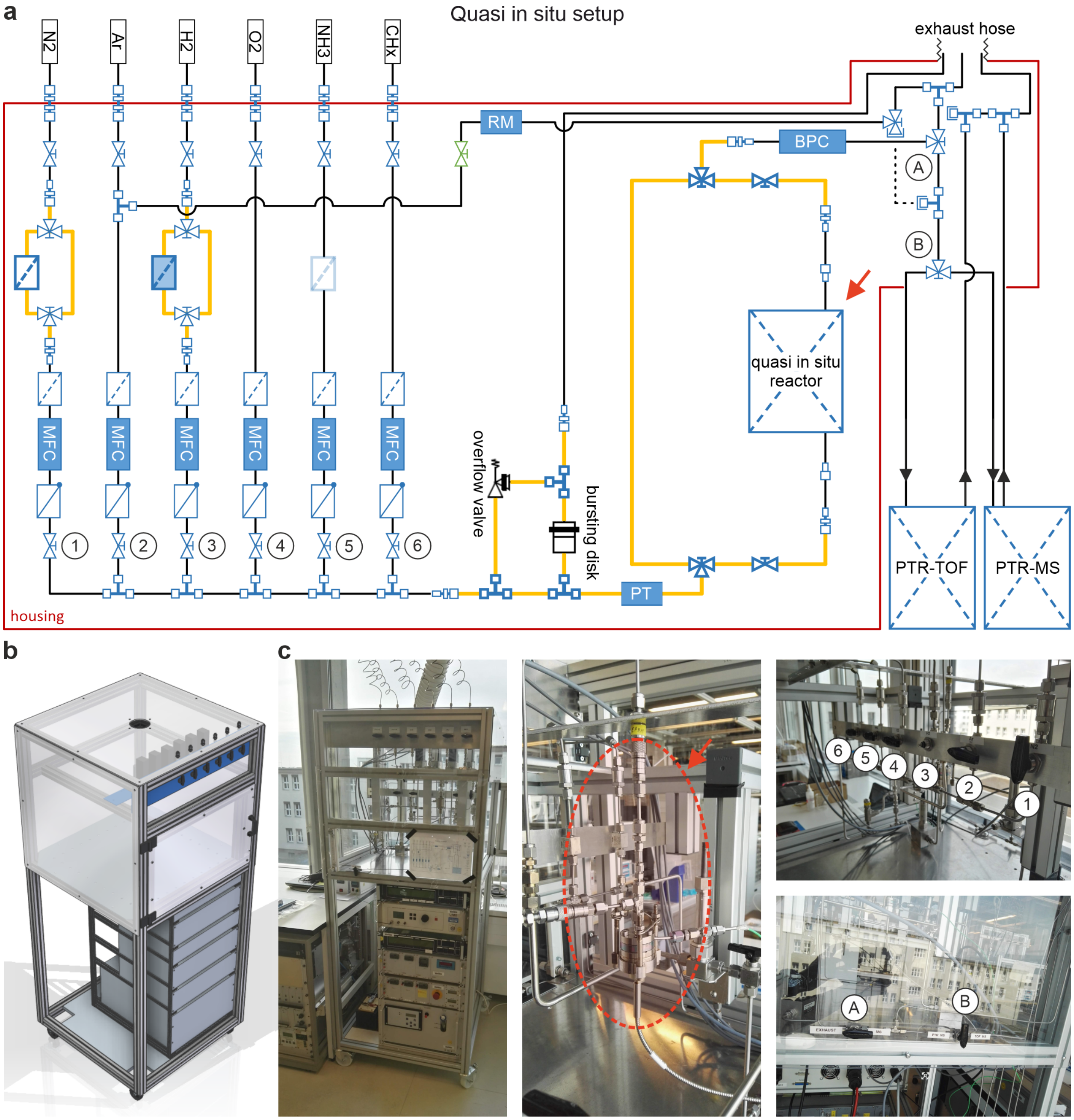

Figure S1: *Quasi in situ* setup. (a) Scheme of the setup design. The individual components are listed in Figure S2. The red arrow points to the TEM-grid reactor, which can be easily mounted and removed *via* quick connectors. (b) Drawing of the rack design. (c) Photo of the rack, with the *quasi in situ* setup in the upper part and the electronic support for mass flow controller, pressure cells and laser in the lower part (right). Magnified view inside the setup with the *quasi in situ* reactor highlighted in red (middle). Gas valves 1-6 for six different gases that can be used and A+B, to switch between the mass spectrometers that are attached to the setup (compare with 1-6 and A+B in (a)).

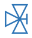	3-way ball valve, 1/8" (SS-41GXS2)	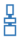	reducing union, 1/4-to-1/8" (SS-400-6-2)	MFC	mass flow controller
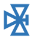	3-way ball valve, 1/4" (SS-42GXS4)	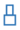	quick connector, 1/8" (SS-QM2-B1-200EP, SS-QM2-D-200EP)	RM	rotameter
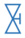	ball valve, 1/8" (SS-41GS2)	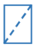	filter, 1/8" (SS-2F-2)	PT	pressure transducer
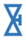	bellows sealed valve, 1/4" (SS-4UW)	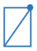	check valve, 1/8" (SS-2C1)	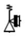	high pressure relief valve, 1/4" (SS-4R3A-EP)
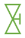	quarter turn valve, 1/8" (SS-2P4T)	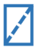	gas purifier N2, 1/4" (MC1-902F)	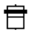	bursting disk
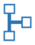	union tee, 1/8" (SS-200-3)	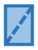	gas purifier H2, 1/4" (MC1-904F)		1/8 inch tubing
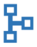	union tee, 1/4" (SS-400-3)	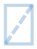	gas purifier NH3 (not yet installed)		1/4 inch tubing
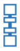	bulkhead union, 1/8" (SS-200-61)	BPC	back pressure controller		1/8 inch tubing (no crossing)

Figure S2: Components that are mounted in the *quasi in situ* setup (compare with Figure S1).

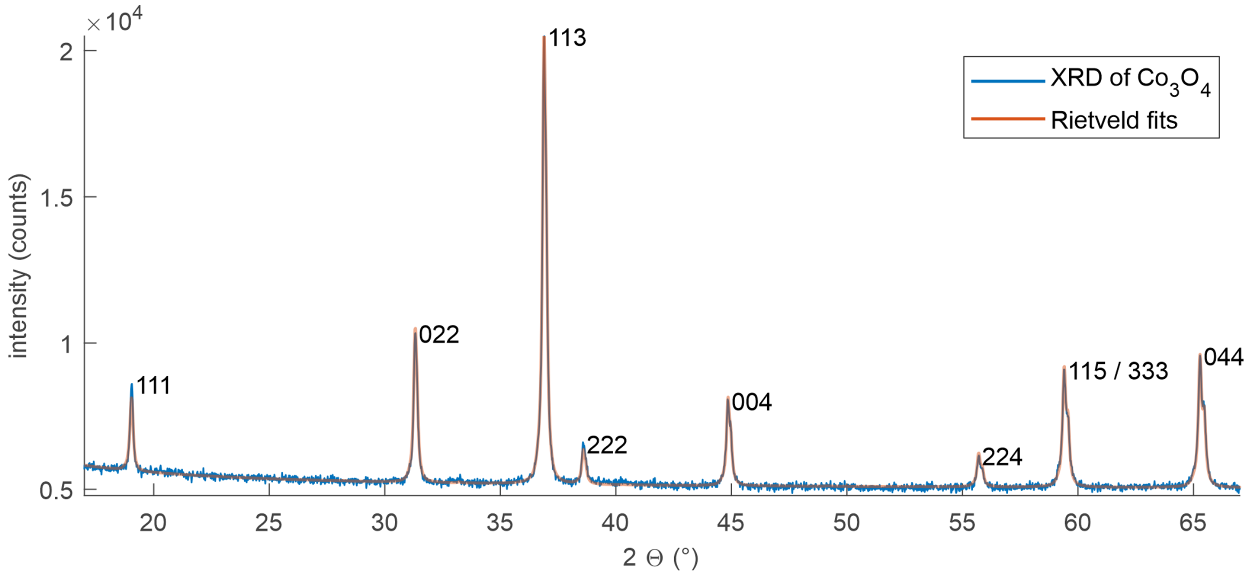

Figure S3: X-ray diffractogram of a powder sample (blue line), showing a phase pure Co_3O_4 spinel structure as verified by Rietveld refinement (red line).

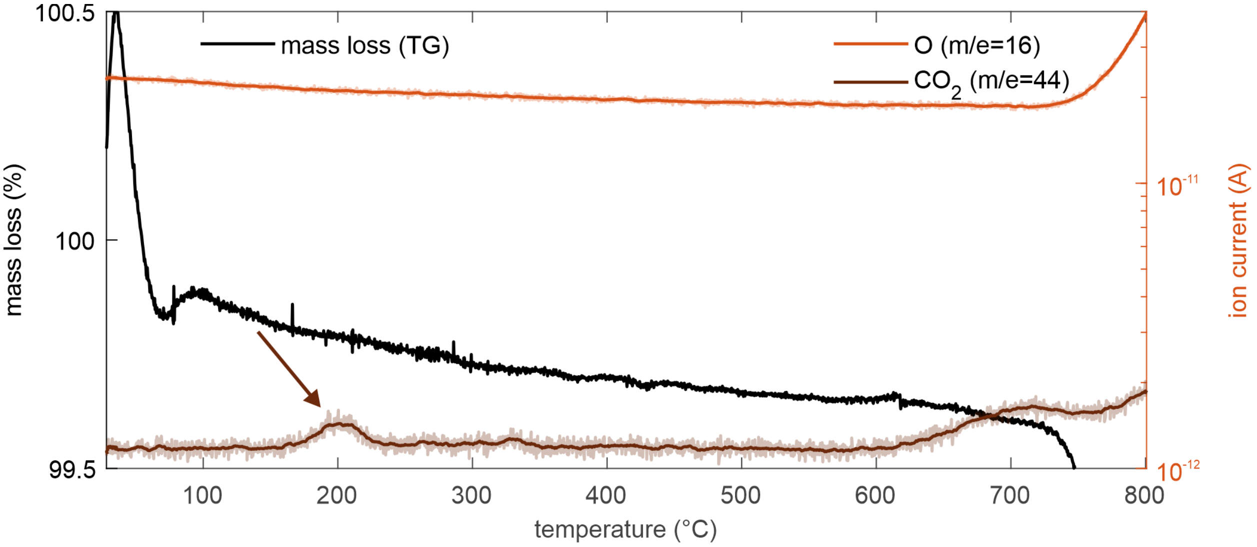

Figure S4: Thermogravimetry (TG) experiments coupled to mass spectrometry (MS). Mass loss measured as function of the heating temperature (black curve, left y–axis), compared with simultaneously measured MS signal (right y–axis) that is associated with oxygen (m/e=16, red curve) and CO_2 (m/e=44, brown curve). The arrow highlight a slight release of CO_2 at around 200 °C from the spinel structure, which starts to decompose at around 750 °C (see black curve, hinting the decomposition together with the increasing release of O (and CO_2)). As the mass loss is low at around 200 °C, we attribute the peak in the CO_2 signal to the thermal decomposition of surface carbonates. Based on this result, we selected a final temperature for the heat treatment at 250 °C to get carbonate-free surfaces.

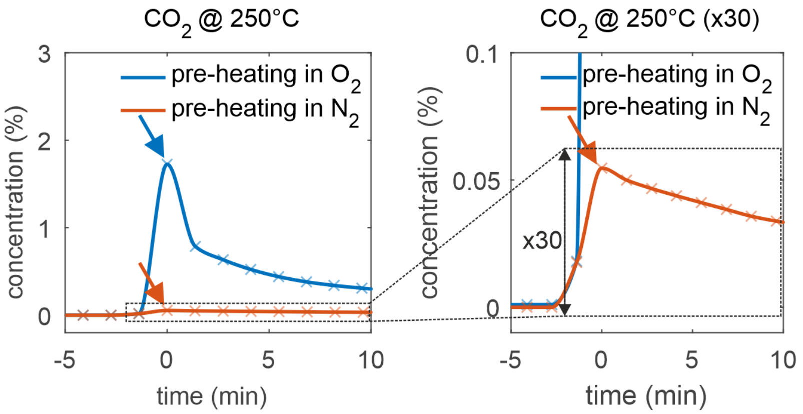

Figure S5: Surface titration using 2 % CO in N_2 at 250 °C for 10 min after a pretreatment at 250 °C for 1 h. CO_2 formation after oxidative (O_2, blue curve) and mildly reductive (N_2, red curve) pretreatments (left) and magnified view of the same data (right). The setup used in this case was exactly the same as used to generate the data shown in Figure 2 of the main text, except that N_2 was used as the reducing agent and gas chromatography (GC) instead of MS.

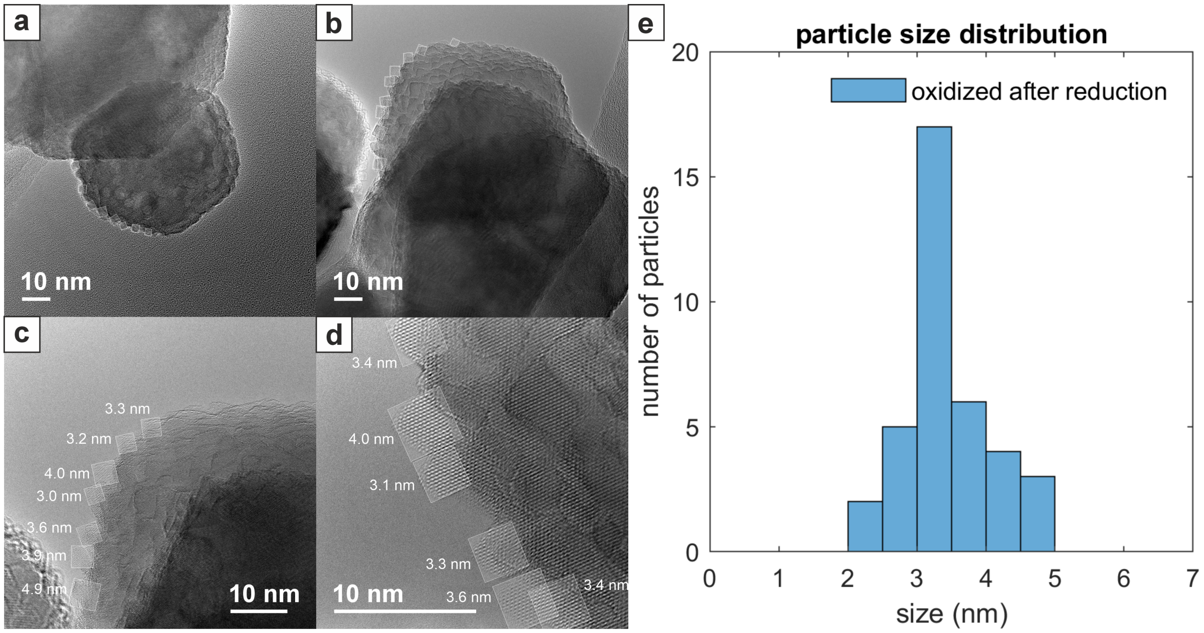

Figure S6: Particle size distribution of cube-like structures at the surface of Co_3O_4 nanoparticles. (a, b) TEM micrographs of two different nanoparticles. (c, d) Magnified views of the particle shown in (b). (e) Particle size distribution of the particles as indicated by white rectangles in (a-d). The particle size refers to the side length of the approximated rectangle/cube.

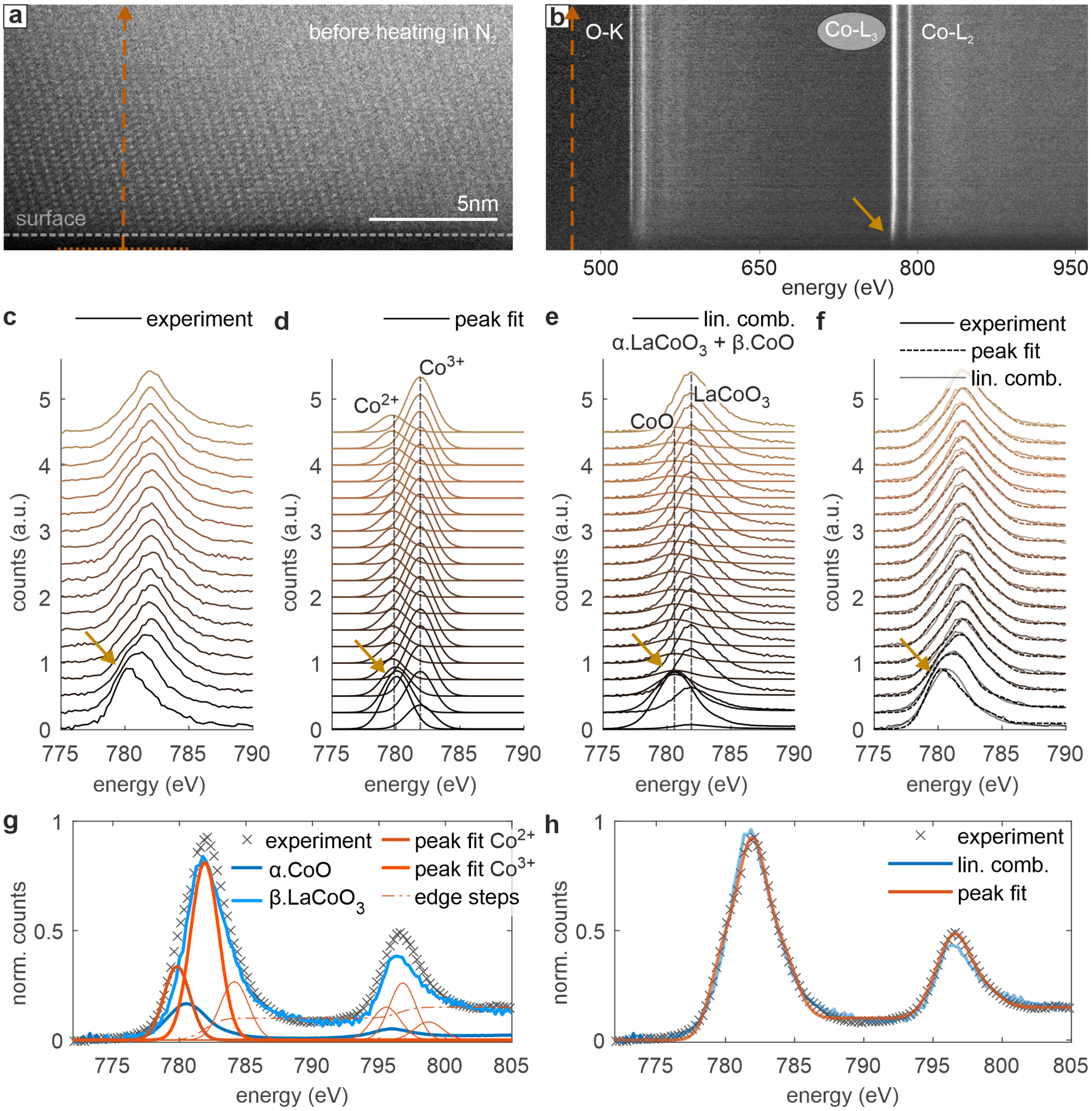

Figure S7: Peak fit and linear combination approach (EELS). (a) Magnified view of the STEM image shown in Figure 4a. (b) 2D view of the deconvolved EELS line scan extracted from the position as indicated by the dashed arrow in (a). The small arrow hints a slight shift to lower energies. (c) Experimental spectra extracted from the line scan in (b). (d) Peak fit approach: Peak fits of the Co^{2+} and Co^{3+} sub peaks. (e) Linear combination approach: CoO and $LaCoO_3$ contributions. (f) Comparison of the experimental spectra with the sum spectra of the peak fit approach and the sum spectra of the linear combination approach shown in (d) and (e). (g) All peaks that contribute to the peak fit approach (red spectra) and the CoO and $LaCoO_3$ contributions for the linear combination approach (blue spectra). The black crosses correspond to the experimental spectrum. (h) Comparison of the experimental spectrum with the sum spectra of the peak fit approach and the sum spectra of the linear combination approach shown in (g).

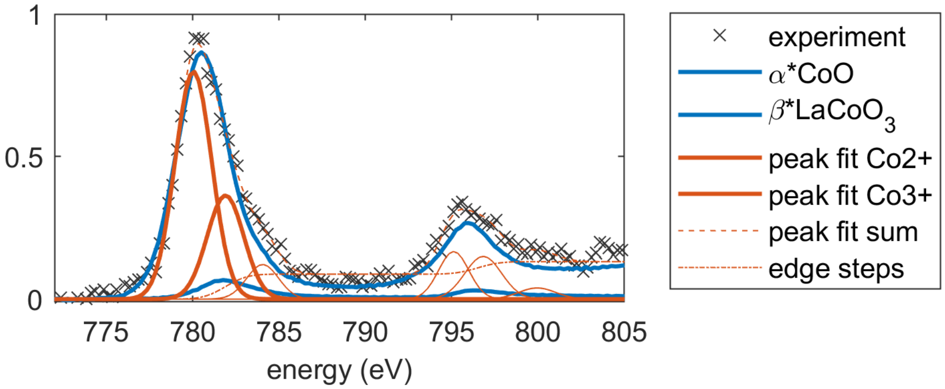

Figure S8: EELS analysis (movie). To play the movie, click the following link for download: https://cloud.fhi-berlin.mpg.de:8443/getlink/fi7KYEiptV8LNgypW2j5oXWi/video_SI.avi

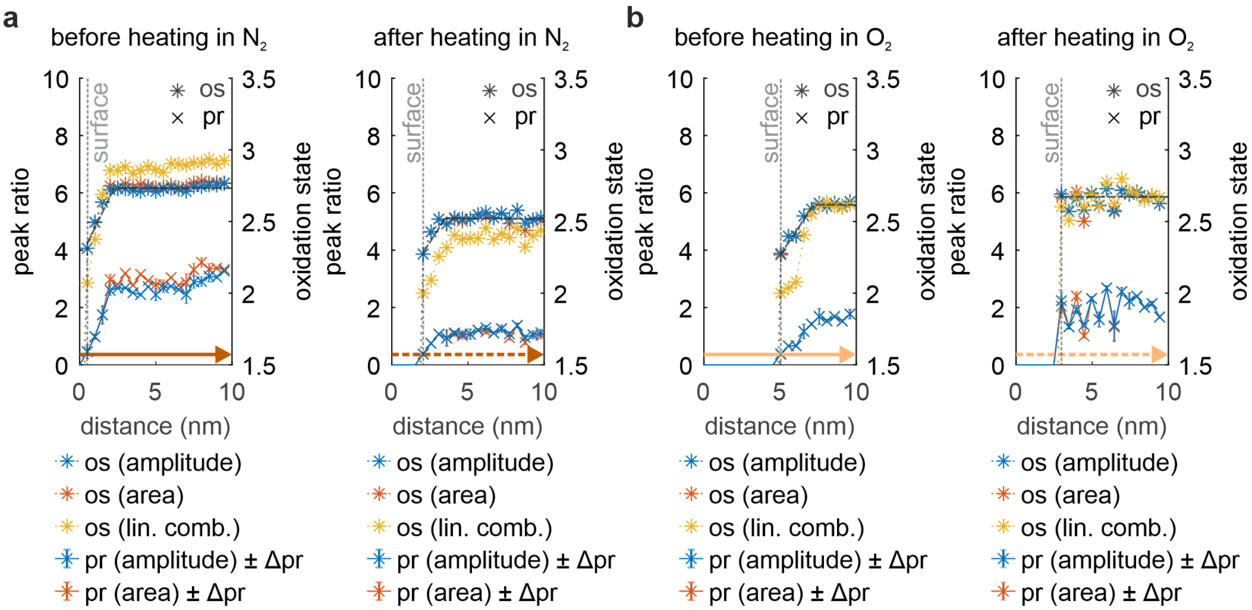

Figure S9: Oxidation state evolution across surface (EELS). (a) Peak ratios (pr) and oxidation states (os) before and after heating in N_2. The vertical dotted lines indicate the surface position of the nanoparticle, the dashed lines are a guide to the eyes. (b) Peak ratios (pr) and oxidation states (os) before and after heating in O_2. The arrows depict the direction of the line scans. This figure corresponds to Figure 5 of the main text, but in addition the following features are shown: (1) The (minor) effect of whether the peak amplitudes or the peak areas (red vs. blue) of fitted Gaussian peaks are used to calculate the peak ratio and from this the oxidation state values. (2) The peak fit approach (blue and red asterisks) shows the same trend as the linear combination approach (yellow asterisk) for the oxidation state values. (3) The fit error Δp (given as 95 % confidence interval) is shown by error bars for "pr (amplitude)" and "p (area)" data points. For further discussion of the periodic fluctuations in panel (b), right plot (after heating in O_2), see Figure S13.

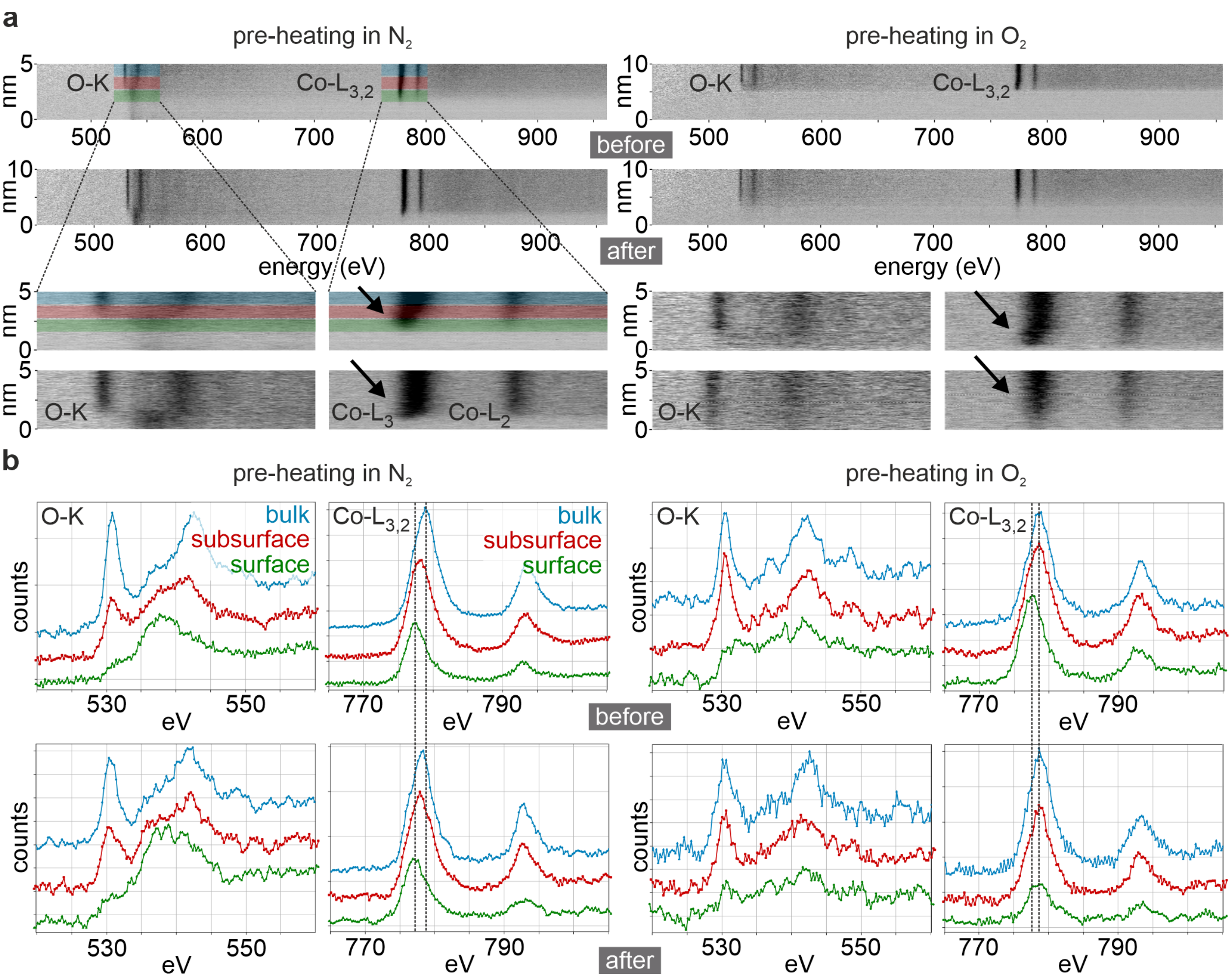

Figure S10: Spectral evolution across interface with conventional software, commercially available (GMS 3.5™, Gatan, Inc.). Although tiny variations in O-K, as well as in the Co-$L_{3,2}$ edge can be resolved, the tracking of the oxidation state failed. (a) 2D view of EEL spectra before and after pre-heating in N_2 (left, top and second row) and pre-heating in O_2 (right, top and second row). Below, a magnified view of the 2D EEL spectra, according to the energy range indicated by the colored areas. The arrows point to the Co L_3 white line. (b) EEL spectra from bulk, subsurface and surface area, extracted from regions as indicated by the colored areas in (a).

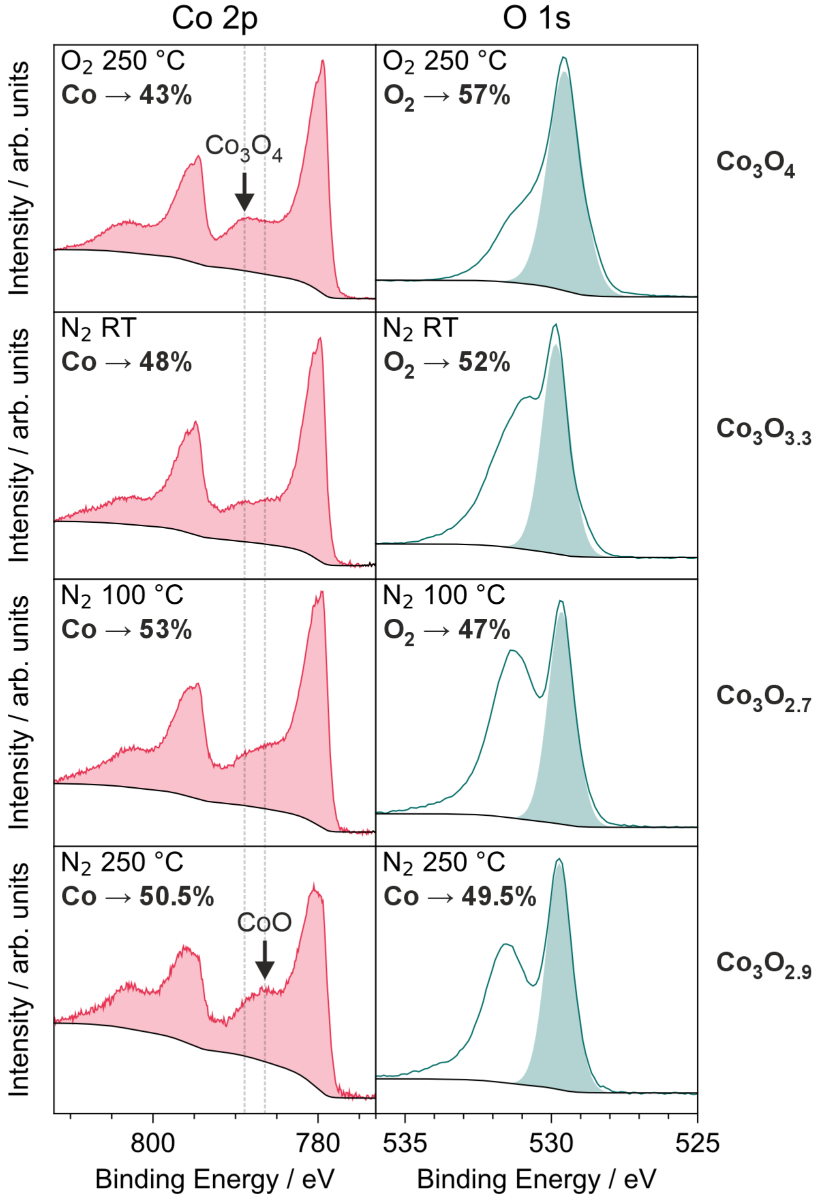

Figure S11: XPS quantification of Co:O ratio. For Co 2p, the whole region was used, for O 1s, only the lattice oxygen peak (shaded peak). While the quantification of the sample oxidized at 250 °C ("O_2 250 °C") shows a stoichiometry of almost Co_3O_4, the sample reduced at 250 °C ("N_2 250 °C") results close to CoO stoichiometry. However, the sample reduced at 100 °C ("N_2 100 °C"), which gives an even lower oxygen content ($Co_3O_{2.7}$), indicates the limitation of the quantification accuracy. In contrast, the Co 2p spectra clearly show a gradual transition toward more Co^{2+} from top to bottom (see vertical dashed lines and black arrows to compare for the relative Co_3O_4 and CoO contributions), with the maximum of the CoO satellite peak at 250 °C ("N_2 250 °C").

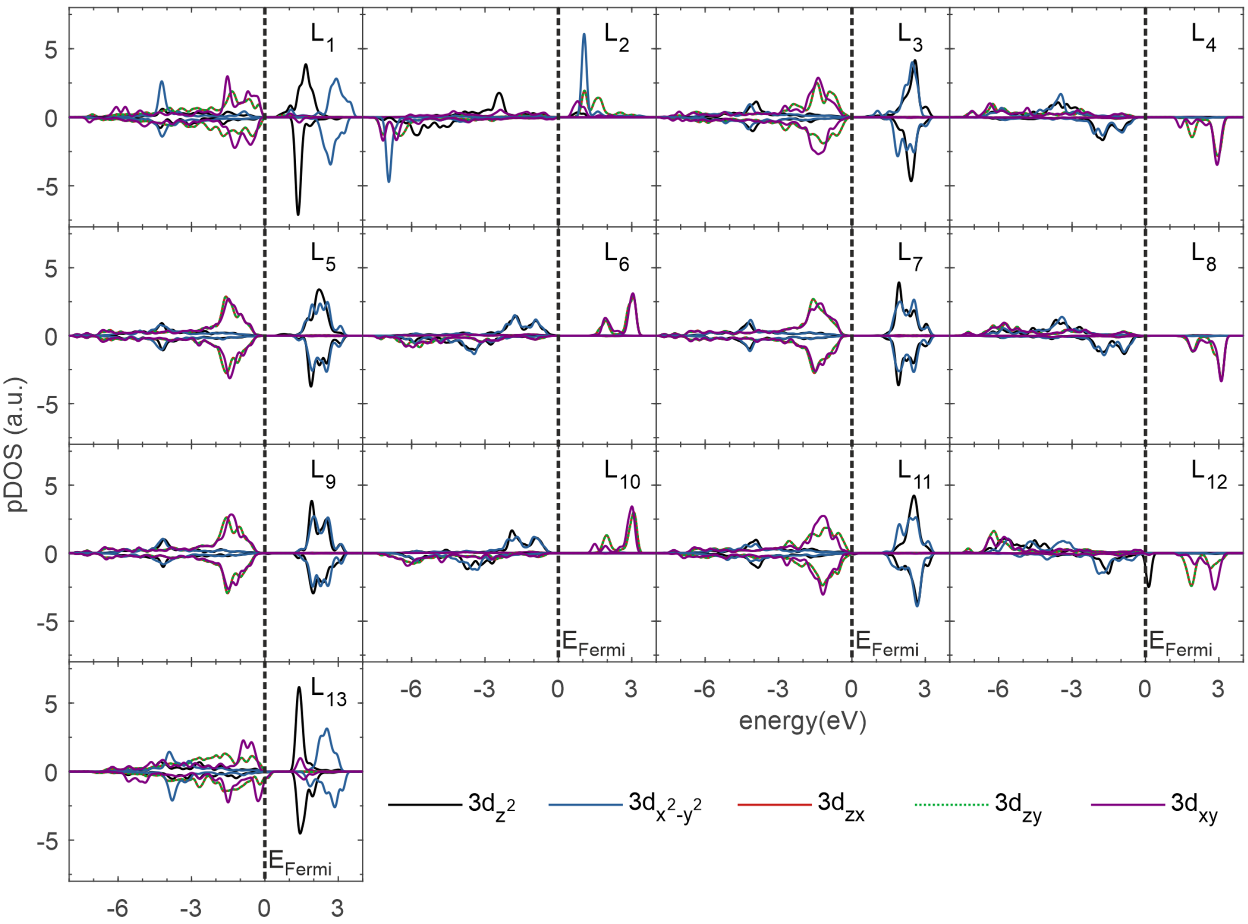

Figure S12: Projected density of states of Co d-states including the occupied pDOS below the Fermi level.

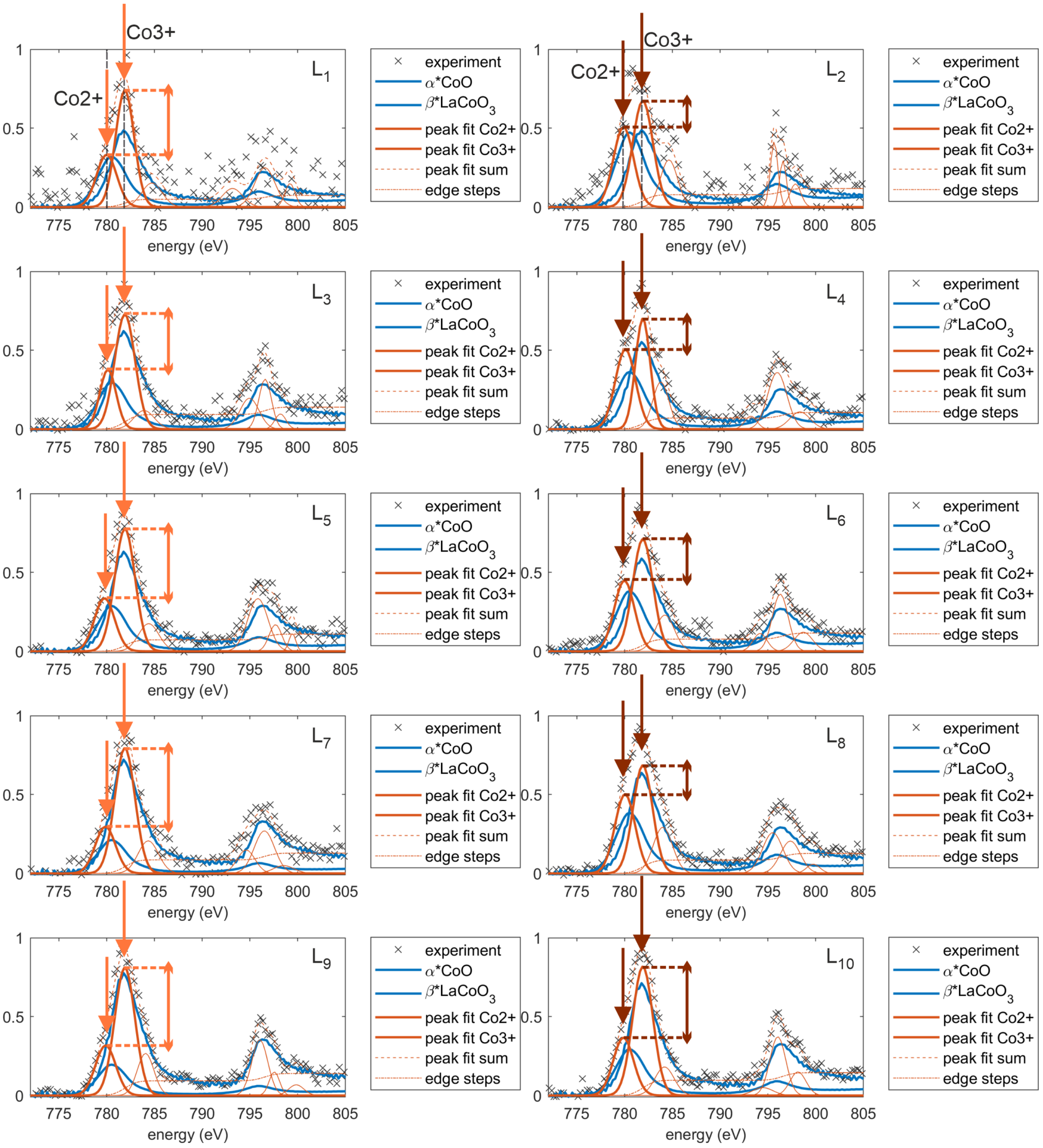

Figure S13: EEL spectra according to Figure 4b, after O_2 heating. L_1 corresponds to the spectra at the surface, L_2-L_{10} are adjacent spectra toward bulk, with a step size of 0.5 nm from layer to layer. The arrows to peak fit Co^{2+} (left) and peak fit Co^{3+} (right) highlight the periodic variation of higher Co^{3+}/Co^{2+} ratio (L_1, L_3, L_5, L_7, L_9) and lower Co^{3+}/Co^{2+} (L_2, L_4, L_6, L_8).

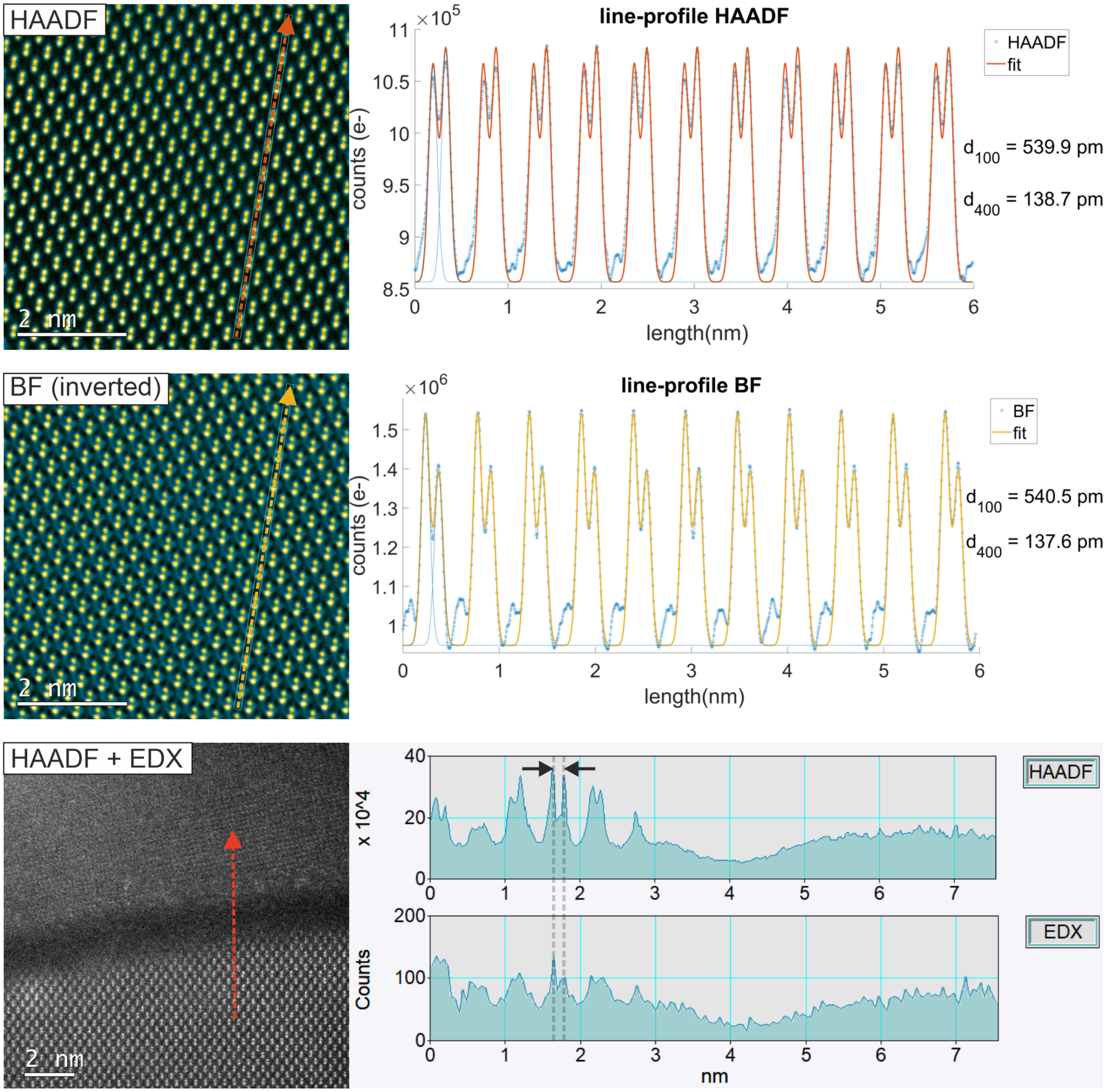

Figure S14: Resolution tests. HAADF and BF STEM images (top, middle - left) and extracted signal profiles (top, middle - right) from Si (110). The BF signal is inverted for better comparability with the HAADF signal. The Si-dumbbells (Si (400)) are clearly resolved, peak fits in red (top) and yellow (middle) result in spacing values close to the expected value of 136 pm. Also the EDX silicon signal (bottom), integrated from 1.65 to 1.85 keV, shows the dumbbells separated together with the simultaneously measured HAADF signal.
